# Selected Molecular Targets for Counteracting Epileptogenesis: What Do We Know About Its Effective Inhibition?

**DOI:** 10.3390/cimb48080842

**Published:** 2026-08-19

**Authors:** Krzysztof Łukawski, Stanisław J. Czuczwar, Barbara Miziak

**Affiliations:** 1 Department of Physiopathology, Institute of Rural Health, 20-090 Lublin, Poland; lukaw@mp.pl; 2 Department of Pathophysiology, Medical University of Lublin, 20-090 Lublin, Poland; stanislaw.czuczwar@umlub.edu.pl

**Keywords:** epileptogenesis, models of epileptogenesis, spontaneous recurrent seizures, neuroinflammation, oxidative stress, epileptogenesis markers

## Abstract

Epilptogenesis is a long-term process that involves the transformation of a healthy brain into a seizure-producing brain. Since approximately 30% of epilepsy patients suffer from drug-resistant seizures, the concept of inhibiting the epileptogenesis process and thus preventing seizures has emerged. The search for effective methods of inhibiting epileptogenesis is possible thanks to animal models, which include kindled seizures; models based on the induction of status epilepticus resulting in subsequent spontaneous recurrent seizures, or brain trauma; and genetic models. Blood–brain barrier dysfunction, inflammatory processes in the brain, and oxidative stress appear to play a major role in epileptogenesis. This prompted testing of a number of anti-inflammatory agents and antioxidants in the epileptogenic process. One noteworthy finding was that losartan (an antihypertensive drug), as a TGF-*β* antagonist, proved effective in inhibiting epileptogenesis due to blood–brain barrier damage. Due to the many mechanisms involved in the process of epileptogenesis, it seems that the use of a combination of drugs will be an effective method of inhibiting it. The most promising combination includes levetiracetam (a second-generation antiseizure drug), atorvastatin, and ceftriaxone (a beta-lactam antibiotic), which effectively inhibits spontaneous seizures in animals experiencing status epilepticus. Any clinical trials on the inhibition of epileptogenesis must take into account the fact that a small percentage of patients develop epileptic seizures after stroke or brain injury. Recently suggested markers predicting a high probability of epileptic seizures after brain damage may facilitate appropriate patient selection for studies on inhibition of epileptogenesis.

## 1. Introduction

Epilepsy, the predominant neurological disease, impacts 65 million individuals globally and imposes significant burdens linked to seizure-related disability, mortality, comorbidities, stigma, and financial expenditures [1]. Despite a significant rise in the availability of antiseizure medications in recent decades, approximately one-third of patients continue to exhibit resistance to medical treatment [1]. Epileptic seizures, resulting from abnormal excessive or synchronous neuronal activity in the brain, are generally classified as either focal or generalized seizures. Focal seizures (partial seizures) arise from networks that are restricted to one cerebral hemisphere and may be distinctly localized or more broadly spread. These seizures can present in multiple forms, with symptoms varying from minor sensory disruptions to more pronounced motor activities and altered states of consciousness. Generalized epileptic seizures begin simultaneously in both hemispheres of the brain and affect cortical and subcortical regions. They typically cause an immediate loss of consciousness or awareness and include absence, tonic, clonic, tonic–clonic, myoclonic and atonic seizures.

For many years, animal models of seizures and epilepsy have played an important role in the development of new antiseizure medications. It is critical in clinical settings that an animal model replicates characteristics of the type of seizure or epilepsy. The more similarities there are in this regard, the more likely it is that an explored antiseizure medication in clinical trials will be effective, as found earlier in the animal model. For example, generalized tonic or tonic–clonic seizures are commonly reproduced using the maximal electroshock seizure model. Temporal lobe epilepsy (TLE) is predominantly represented by kindling models (the amygdala or hippocampal kindling), as well as chemoconvulsant models like pilocarpine (PILO)-induced or kainic acid (KA)-induced epilepsy. Since the subject of the present review concerns the phenomenon of epileptogenesis, the most important animal models of epileptogenesis are described in the next chapter.

The term “epileptogenesis” describes the development and extension of tissue that can produce spontaneous recurrent seizures (SRSs), which can lead to either the onset of an epileptic condition or the progression of epilepsy once it has been established [2]. Basically, epileptogenesis involves three stages: (1) the initial precipitating insult (such as, e.g., traumatic brain injury), (2) the latent period, which can last weeks to months and is characterized by cellular and molecular changes that affect the neuronal network’s excitability, and (3) the chronic epilepsy phase, during which SRSs are experienced [3]. It has been shown that epileptogenesis is progressive with continuing changes in the neuronal network extending into the chronic epilepsy period [3]. In other words, this process is responsible for the onset, development, and progression of epilepsy [4]. As a result, inhibiting epileptogenesis is an important research area with the potential to bring significant benefits to patients by avoiding the medical and social implications of epilepsy and lifetime treatment [5]. The therapeutic window of intervention has been expanded to cover both disease prevention and improvement of its clinical course due to the evidence of progressive alterations and increasing seizures [4]. Antiepileptogenesis is regarded as a process that counteracts the effects of epileptogenesis, encompassing prevention, seizure modification, and cure; antiepileptogenic agents can be administered prior to or after epilepsy onset [2].

The following conditions have been suggested to induce epileptogenesis: traumatic brain injury (TBI), stroke, infections, tumors, cerebral palsy, craniotomy, neurodegenerative diseases, intrapartum hypoxia, or prolonged acute symptomatic seizures, including complex febrile seizures and status epilepticus (SE) [5,6]. These inciting insults can lead to cellular and structural alterations that may occur in the epileptogenic brain and include neurodegeneration, gliosis, neurogenesis, dendritic plasticity, mossy fiber sprouting, recruitment of inflammatory cells into brain tissue, damage to the blood–brain barrier (BBB), reorganization of the extracellular matrix or the molecular architecture of specific neuronal cells, and epigenetic changes [7]. Certain alterations seem to plateau once chronic seizures are established (such as neuronal cell loss, mossy fiber sprouting, and neurogenesis), while others (including glial cell plasticity, BBB dysfunction, transcriptomic changes, and epigenetic modifications) are dynamically affected by the onset and recurrence of seizures, thereby contributing reciprocally to the seizures and creating a pathological vicious cycle [4]. The specific mechanism by which an initial brain insult induces epileptogenesis remains uncertain. The identification of molecular and functional gene and/or protein networks impacted by an epileptogenic insult or subsequent epileptogenesis was made possible by in-depth analyses of large data sets, resulting in the development of hypotheses regarding the molecular alterations that may be pertinent to epileptogenesis [2]. Proteolytic cascades, TGF-β (transforming growth factor β) and IGF-1 (insulin-like growth factor 1) signaling, inflammatory cytokines, p38 MAPK (p38 mitogen-activated protein kinase), JAK/STAT (Janus kinase/signal transducers and activators of transcription), PI3K (phosphatidylinositol 3-kinase), mTOR (mammalian target of rapamycin), complement activation, and gene expression modulation related to glial oxidative stress all play a role in epileptogenesis [2,8]. Most of these mechanisms have been studied in animal models of epileptogenesis. In this review, we do not attempt to provide a general overview of epileptogenesis but rather limit ourselves to discussing selected mechanisms that, in our opinion, constitute the basis of epileptogenesis research, presenting substances that may play a significant role in inhibiting or preventing epileptogenesis.

## 2. Search Strategy and Selection Criteria

PubMed, Google Search, and Web of Science databases were searched for relevant article using the following keywords: epileptogenesis and mechanisms of action, epileptogenesis and animal models, epileptogenesis and neuroinflammation, epileptogenesis and oxidative stress, epileptogenesis and blood–brain barrier dysfunction, epileptogenesis and inhibition, epileptogenesis and markers. No restrictive date limits were applied. Some of the references come from selected articles. This is a narrative review, genetic/epigenetic mechanisms were excluded, and no predefined inclusion/exclusion criteria were used.

## 3. Models of Epileptogenesis

Epilepsy research aims to develop antiepileptogenic including disease-modifying treatments. Numerous therapeutic options have been developed during the last decades, and many of them have demonstrated significant potential in animal models. In this chapter, we discuss the primary animal models of epileptogenesis including their limitations.

Basically, the kindling model, in which rats’ limbic system (amygdala or hippocampus) is subjected to repeated electrical stimuli, as well as post-SE rodent models, primarily PILO and KA models, have been widely used in epileptogenesis studies [9]. When the same stimuli are repeated in the kindling model, the seizures’ duration and intensity gradually increase, which is the acquisition of kindling [6]. Fully kindled seizures have characteristics akin to complex partial seizures with secondary generalization, so amygdala or hippocampal kindling is regarded as a model of TLE [6,10]. Prolonged daily kindling over several weeks and months (“overkindling”) results in spontaneous convulsive seizures in around fifty percent of rats, signifying an extended latent phase [6,10]. However, there are arguments pointing to the weaknesses of kindling as a model of epileptogenesis. It is not fully understood to what extent epileptogenesis occurs during kindling and which type of epileptogenic brain insult it represents. It has been suggested that kindling with brain damage induced by depth electrode implantation may provide a model in which the effects of TBI are exacerbated by electrical stimulation [6]. Furthermore, kindling can only simulate secondary epileptogenesis—not primary—because animal models do not produce spontaneous seizures before kindling. The applicability of kindling to human epilepsy is still up for debate because there are no analogies in the human situation outside of extremely rare observations with deep brain stimulation [11]. As a sequel to traditional kindling, chronic electrical stimulation using corneal electrodes was introduced in mice as a way to obtain in shorter time large numbers of kindled animals that exhibit seizure phenomena reflecting partial seizures in humans [12]. This model is currently available as one of the models of epileptogenesis [13]. According to some authors, insufficient persistence of corneal kindling in mice detracts from the use of this model for repeated drug testing in the same group of animals [14].

In addition to the electrical kindling model, the chemical kindling method using pentylenetetrazole (PTZ), a GABA-A receptor antagonist, is used in epileptogenesis research and deserves mention due to its wide application. Treatment with PTZ (25–40 mg/kg) via the intraperitoneal (i.p.) or subcutaneous (s.c.) route every other day induces kindling in over 80% of mice [15]. The antiepileptogenic effect of any specific drug or therapy is evaluated by measuring the latency to myoclonic jerks, the frequency of myoclonic jerks, the latency to the onset of generalized tonic–clonic seizures, and the number and severity of generalized tonic–clonic seizures observed after each subconvulsant PTZ injection during kindling [2]. The primary limitation of this model is that it relies on a specific chemical trigger, which does not truly reflect the many and multifaceted causes of epilepsy in reality.

In particular, SE-based models have gained a prominent role in epilepsy research in recent years. They stem from the observation that injections of specific chemoconvulsants or repetitive electrical stimulation of certain brain structures can lead to a chronic epileptic state with robust convulsive SRSs [2]. In terms of seizure phenomenology, electroencephalographic (EEG) characteristics, cognitive results, and neuropathology, PILO and KA have been the most thoroughly studied of the many systemic chemoconvulsants. Both models exhibit partial SRSs and secondarily generalized seizures in rats, alongside hippocampal and extrahippocampal damage, as well as behavioral and cognitive changes that mirror the clinical features of TLE [16]. In PILO and KA models, SE is usually terminated after 60 to 90 min (sufficient time to induce epilepsy in most rats or mice) using diazepam or general anesthetics to reduce the mortality rate [6]. In models where SE is caused by focal electrical stimulation of the hippocampus or amygdala, 3–4 h of SE are required to induce epileptogenic activity. However, the chemoconvulsant-induced SE is more severe than electrically generated SE and is more difficult to terminate with diazepam [6]. Because SE models are induced in healthy animals without an underlying etiology (uncommon for SE in humans), their usage may result in translational problems [17]. Furthermore, the majority of animals in PILO and KA models that receive systemic administration of high doses of these convulsants develop epilepsy, which is linked to extensive brain damage and a nearly nonexistent latent period between SE and the onset of seizures. As drug testing in such models takes place in the latent period, their sensitivity to detect antiepileptogenic drugs may be low [17].

Preclinical investigations on epileptogenesis are optimally conducted using an animal model where the timing of a potentially epileptogenic insult to a normal brain is established, allowing for the monitoring of the epileptogenic process from injury to the onset of epilepsy. A suitable candidate clinical disease that meets these criteria for modeling is TBI [18]. Indeed, a number of models of posttraumatic epilepsy (PST) have been introduced. The existing data indicate that both focal contusion and TBI accompanied with grey and white matter damage can lead to epileptogenesis [2]. Additionally, animal models must also have translational potential, which is an advantage when it comes to TBI models [18]. The same applies for models of poststroke epilepsy. However, the frequency of seizures during the monitoring period and the percentage of animals that develop epilepsy in models of poststroke epilepsy have varied, and it is unclear if this variability is due to differences in the severity of the injury or other experimental factors [2]. Similar to TBI models, epileptogenesis usually seems to be delayed and limited to a percentage of animals, and seizure frequency is low [2].

Epileptogenesis also functions in genetic epilepsy [19]. Important information on the role of certain mutations in epileptogenesis can be gained from genetic models of epilepsy. Additionally, many of these models are turning into valuable resources for confirming novel epilepsy treatment and prevention targets [17]. Briefly, the most commonly used models include knockout (when a specific gene is inactivated) and knockin models that introduce mutations in epilepsy-related genes, e.g., *SCN1A*, *SCN2A*, *SCN8A*, *KCNA1*, *CACNA1A*, *CACNG2*, *GABRA1* and *GRIN2A*. Animals with these mutations exhibit SRSs, abnormal brain electrical activity, and developmental changes resembling the clinical features of human epilepsy. Models of central nervous system (CNS) channelopathies are particularly valuable because they allow for the analysis of the impact of Na^+^, K^+^, and Ca^2+^ channel dysfunction on the development of epileptogenesis. For a review on genetic models of epilepsy, see [20].

## 4. Blood–Brain Barrier Dysfunction as a Key Factor in Primary Epileptogenesis?

Antiepileptogenic therapies may address the dual aspects of epileptogenesis: (1) primary epileptogenesis, the shift from a brain state devoid of unprovoked seizures to one characterized by their occurrence, and (2) secondary epileptogenesis, wherein preexisting epilepsy intensifies over time [21]. Preserving the integrity of the blood–brain barrier (BBB) is crucial for ensuring an optimal environment for nervous tissue function. Evidence indicates that BBB disruption may precipitate epileptic seizures, whereas conversely, seizure-induced BBB breakdown may lead to more epileptic episodes [22]. In their study, Mendes et al. [22], using a PILO-induced SE model of epileptogenesis, demonstrated that BBB breakdown is a dynamic phenomenon and time-dependent. PILO-induced alterations in brain tissue initially enhance BBB permeability to micromolecules, followed by a macromolecule leakage approximately 5 h after SE. Although the leakage of macromolecules is terminated 24 h after SE, BBB permeability to micromolecules continues and the effects of BBB dysfunction are broadly distributed throughout the brain, which may lead to additional BBB breakdown events. This and other findings indicate that BBB leakage occurs during epileptogenesis and the chronic epileptic phase [23]. The specific mechanisms underlying BBB disruption remain to be completely elucidated; however, it seems that paracellular leakage due to the dysfunction or downregulation of tight-junction proteins, along with increased transcellular transport across the endothelial barrier, may significantly contribute here [24]. The pathological changes may vary depending on different disease conditions. Generally, BBB disruption results in a chain of processes including ion dysregulation, oedema, and neuroinflammation, potentially leading to neuronal dysfunction, elevated intracranial pressure, and neuronal degeneration [25]. Leukocyte infiltration, accumulation of blood proteins including albumin and fibrinogen, and the ensuing microglial activation and astrogliosis are all signs of BBB disruption [25]. As the first stage, the proposed epileptogenic cascade initiated by BBB dysfunction encompasses the cross-BBB influx of albumin from serum into the brain neuropil [24]. In their work, Seiffert and colleagues [26] were the first to use a model of focal disruption of the BBB in the rat cerebral cortex by direct administration of bile salts. In vivo exposure of the cerebral cortex to bile salts resulted in sustained extravasation of serum albumin into the extracellular space of the brain and was associated with marked astrocyte activation leading to epileptic activity. The authors suggested that exposure of the serum-free brain environment to serum proteins underlies epileptogenesis in the cerebral cortex with a damaged BBB and may play a role in the pathogenesis of focal epilepsy. Subsequent research has demonstrated that the administration of the blood-borne protein albumin, either through direct perfusion onto the cortex or via injection into the ventricles, was adequate to induce a delayed onset of SRSs and lower the seizure threshold in both epileptic animals and those subjected to PTZ-induced seizures [24]. Later, it was discovered that the albumin loading of cerebral glia is facilitated by a particular receptor for transforming growth factor β (TGF-β), a potent regulator of apoptosis and the cell cycle [27]. TGF-β transmits signals by interacting with two distantly related transmembrane serine/threonine kinases known as receptors I and II. TGF-β binds to receptor II (TβRII), which subsequently phosphorylates the TGF-βI receptors (TβRI), referred to as activin-like kinase 5 (ALK5) [28]. The TGF-β isoforms (TGF-β1, -β2 and -β3) share common structural elements but show different binding affinities for TβRII. The TGF-β signaling system is relayed by Smad proteins and non-Smad pathways (Erk, JNK, p38 MAPK, etc.), which regulate context-specific gene responses and therefore control different cellular processes [29]. ALK5 phosphorylates intracellular mediators Smad 2 and 3 [30]. Albumin binding to the astrocytic TGFβ receptor ALK5 and its activation followed by the enhancement of inflammatory signaling, constitute the next steps in the proposed cascade for epileptogenesis [24]. A variety of modifications transpire in astrocytes with exposure to albumin, which subsequently modifies their function and affects the surrounding neurons. Albumin uptake by astrocytes via TGF-β receptors induces elevated intracellular calcium levels, which downregulate inward rectifying potassium (Kir 4.1) and aquaporin-4 (AQP4) channels, diminishing extracellular potassium buffering and promoting NMDA-mediated neuronal hyperexcitability [22]. Further, it has been demonstrated in vitro and in vivo that activation of the astrocytic ALK5/TGF-β-pathway induces excitatory, but not inhibitory, synaptogenesis that precedes the appearance of seizures [31]. TGF-β signaling activation in astrocytes also participates in degradation of perineuronal nets around GABAergic interneurons [32]. These alterations enhance the imbalance between excitation and inhibition, as well as augment neuronal synchronization, resulting in the emergence of epileptiform activity, followed by SRSs [24]. Consequently, transcriptional changes in astrocytes resulting in astrocytic decoupling, diminished potassium and glutamate buffering capacity, degradation of the extracellular matrix, and the proliferation of new excitatory synapses, along with the reorganization of neural networks to enhance excitatory activity, represent subsequent phases in the cascade of epileptogenesis triggered by BBB disruption [24]. There is evidence that, in addition to astrocytes, neurons can absorb albumin [33]. Although the presence of albumin in neurons can lead to death, it seems that albumin per se is not neurotoxic [22]. However, overwhelming scientific data demonstrate the link between loss of BBB function and the specificity of albumin accumulation in astrocytes, as mentioned above.

**Figure 1:**
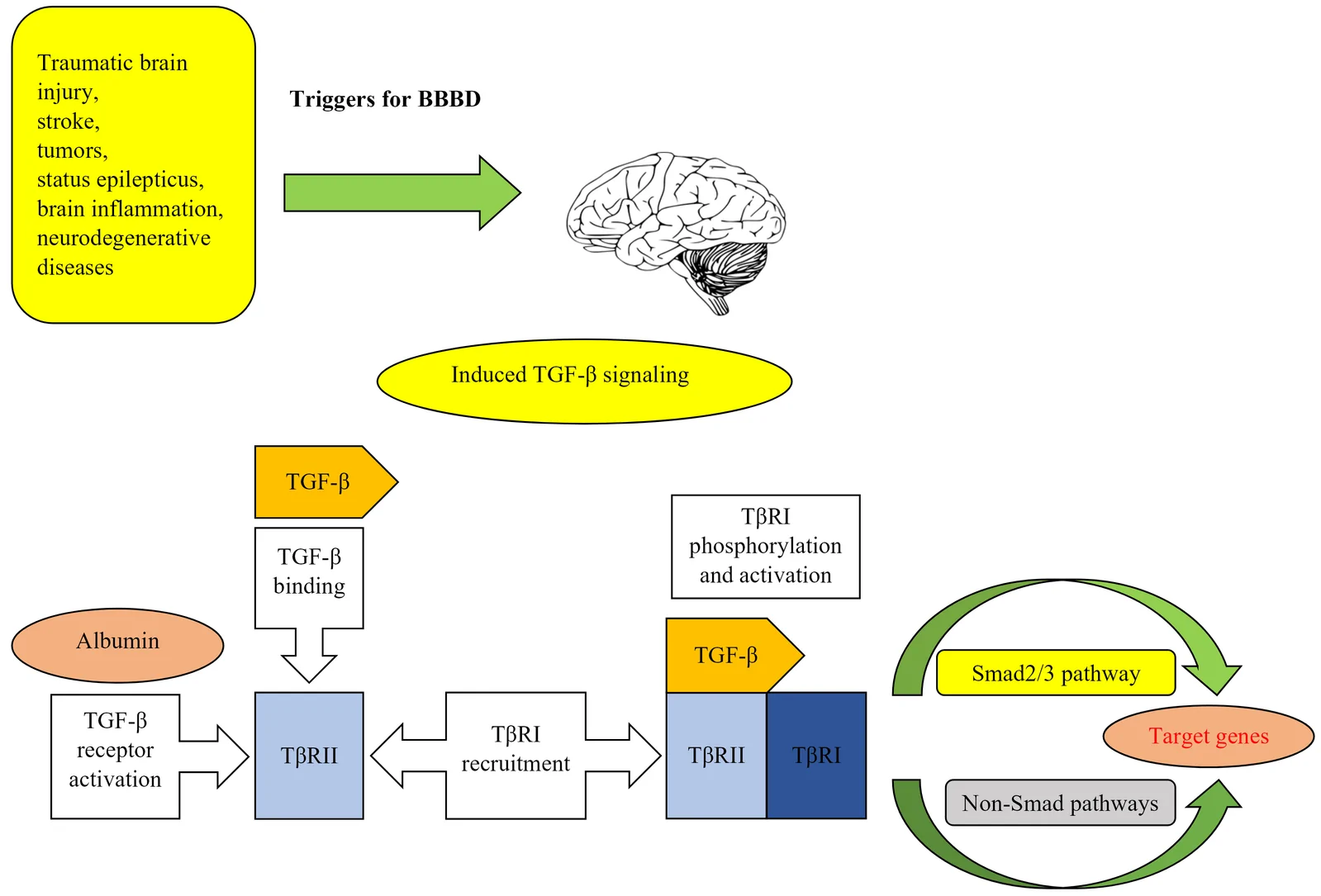
Blood–brain barrier dysfunction (BBBD)-induced inflammatory TGF-β signaling. BBBD has been shown in both patients and animal models across various pathological conditions including traumatic brain injury, stroke, brain tumors, status epilepticus, brain inflammation or neurodegenerative diseases. Albumin functions as a signaling molecule that initiates an inflammatory response to damage [24]. TGF-β binds directly to receptor II (TβRII), which is a constitutively active kinase. Bound TGF-β is subsequently acknowledged by receptor I (TβRI), which is incorporated into the complex and undergoes phosphorylation by TβRII. Phosphorylation enables TβRI to transmit the signal to downstream substrates [28]. The TGF-β signaling system is relayed by Smad and non-Smad pathways (Erk, JNK, p38 MAPK, etc.), which regulate context-specific gene responses and hence control diverse cellular processes [29].

A suitable example illustrating the relationship between BBB dysfunction and epileptogenesis may be the pathological changes occurring in the brain and the clinical consequences of COVID-19. Many studies reported that patients with no history of epilepsy or other neurological conditions developed new-onset refractory SE (NORSE) weeks, months, or even up to a year following SARS-CoV-2 infection [34]. The direct effect of the SARS-CoV-2 spike protein (S1-protein) and pro-inflammatory cytokines (IL-1β, IL-6, and TNF-α) secreted by host immune cells disrupts the integrity of the BBB, facilitating viral penetration through the endothelium and subsequent infection of astrocytes [35]. The infiltration of viruses into the CNS triggers the secretion of pro-inflammatory cytokines, prostaglandin E2, nitric oxide (NO), and free radicals, resulting in persistent inflammation, neural hyper-excitability, seizures, and death [36]. The primary reason of COVID-19’s devastating effects on the CNS is a cytokine storm that is either created by pro-inflammatory cytokines produced by activated microglia or peripheral cytokines entering the CNS due to BBB breakdown [36]. Clinical consequences of COVID-19 could include acute and secondary seizures. NORSE can manifest following post-COVID complications such as stroke [34]. The BBB can be disrupted by endothelial cell damage after stroke, which allows albumin to enter the CNS and bind to TGF-β receptors on astrocytes, activating TGF-β signaling. This is followed by a decrease in the expression of the Kir 4.1 potassium channel and the glutamate transporter. As a result, the concentration of potassium and glutamate in the synaptic cleft increases and can cause seizures [36]. Consequences of BBB dysfunction are shown in Figure 1.

## 5. Neuroinflammation

The dysfunction of the BBB often results from inflammatory changes, whether related to trauma or not, suggesting a relationship among inflammation, BBB, and seizures [37]. 

A growing body of experimental data indicate that inflammation plays an important role in the pathophysiology of seizures and epileptogenesis. Using both in vitro and in vivo experimental models, research revealed some important findings: (1) SE-induced brain inflammation intensifies during epileptogenesis; (2) brain inflammation increases neuronal hyperexcitability and seizures; and (3) inflammation associated with seizures or injuries may contribute to cell loss and synaptic reorganization, which are crucial mediators of the development of hyperexcitable circuits that result in epilepsy following insults like SE or TBI in the adult rodent brain [38].

Neurons and astrocytes respond to brain damage through the release of proinflammatory signals. Upon cellular activation, glial cells produce and secrete several neural mediators, such as interleukin-1β (IL-1β), tumor necrosis factor-α (TNF-α), interleukin-6 (IL-6,) chemokines CXCL8/IL-8, CXCL1 and CCL2, and protein HMGB1, whereas hyperactive neurons generate cyclooxygenase-2 enzyme (COX-2) and release prostaglandins, HMGB1, ATP, and fractalkine [39]. This section of the review examines inflammatory pathways considered essential to the molecular mechanisms underlying epilepsy.

### 5.1. Inflammatory Cytokines

#### 5.1.1. Interleukin-1β

Inflammatory cytokines, including IL-1β, TNF-α, and IL-6, have been demonstrated to be overexpressed in experimental seizure models within brain regions responsible for seizure production and propagation, mostly by glial cells [8]. IL-1β, synthesized by glia (microglia and astrocytes), endothelial cells of the BBB, and leukocytes, is regarded as the principal cytokine in epileptogenic brain tissue [38]. Apart from IL-1β, seizures and epileptogenic brain lesions in animal models increase the expression of IL-1β receptor type 1 (IL-1R1) and endogenous IL-1β receptor antagonist (Ra) [39]. The stimulation of the IL-1β–IL-1R1 axis facilitates convulsions while its inhibition has a protective effect on seizure production. Intrahippocampal injection of IL-1β before KA application enhanced KA-induced seizures. This effect was blocked by co-injection of a IL-1β receptor antagonist [40], indicating that exogenously applied IL-1β prolongs seizures in an IL-1R1-mediated manner [41]. Additionally, it has been documented that intrahippocampal administration of recombinant IL-1Ra or its selective endogenous overexpression in astrocytes regulated by the glial acidic fibrillary protein promoter significantly suppresses seizures produced by bicuculline in mice. Moreover, recombinant IL-1Ra was ineffective in mice deficient in IL-1R1. It has been proposed that a crucial mechanism to control seizure generalization is the equilibrium between brain IL-1β and IL-1Ra [41]. The blockade of IL-1β synthesis in the brain with IL-1β converting enzyme (ICE)/Caspase-1 inhibitor (VX-765) provided further evidence of the involvement of IL-1β in the generation of seizures. In a model of acute KA-induced convulsions, administration of VX-765 to rats inhibited seizure-induced production of IL-1β in the hippocampus [42]. Furthermore, VX-765 treatment resulted in a delay in seizure onset and significant reduction in seizure duration both in rats and mice [42,43].

It has been documented that IL-1β is highly elevated in activated microglia and astrocytes during both the acute phase of SE and in the chronic phase of SRSs in epileptogenic brain regions [44]. The likelihood that IL-1β is implicated in the mechanisms generating SRSs is suggested by the persistent expression of IL-1β during epileptogenesis, in contrast to other cytokines whose increase is restricted in time to current epileptic activity [8]. The role of the IL-1β pathway in epileptogenesis has been examined through: (1) assessing the impact of VX-765 on the progression of hippocampal kindling; (2) employing pharmacological or epigenetic interventions during the interval between the initial injury (typically SE) and the anticipated onset of the disease, or transiently following the onset of epilepsy; (3) analyzing epilepsy development in transgenic mice deficient in particular signaling molecules [44].

An investigation into the molecular processes responsible for the pathological effects induced by the activation of the IL-1β–IL-1R1 axis, uncovered a functional connection between IL-1β and glutamatergic as well as GABAergic neurotransmission. The functional consequences for the association of IL-1R1 with the NR2B subunit of the NMDA receptor have been found. IL-1β activates IL-1R1 in primary cultures of rat hippocampal neurons, leading to fast MyD88-dependent phosphorylation of Src protein kinases and subsequently NR2B tyrosine phosphorylation [45]. A primary outcome of this signaling is the IL-1β augmentation of NMDA-mediated neuronal Ca^2+^ influx, leading to increased excitotoxicity [45]. The proconvulsive effect of IL-1β, contingent upon the activation of a sphingomyelinase- and Src-family kinase-dependent pathway in the hippocampus, resulting in the phosphorylation of the NR2B subunit, has been demonstrated in vivo during experimental seizures [46]. Furthermore, IL-1β was demonstrated to inhibit NMDA-induced outward currents through IL-1R1 in hippocampal CA1 neurons involving p38 mitogen-activated protein kinase (p38 MAPK) and the phosphorylation of Ca^2+^-dependent K+ channels [47,48]. It has also been reported that IL-1β selectively regulates phosphorylation and surface expression of the alpha-amino-3-hydroxy-5-methyl- 4-isoxazolepropionic acid (AMPA) receptor subunit GluR1, dependent on extracellular Ca^2+^ and NMDA receptor activation [49]. Some studies indicate that IL-1β diminishes GABA-A-mediated inhibitory neurotransmission, which may potentially contribute to the occurrence of seizures. IL-1β administration to rat hippocampal slices decreased synaptic inhibition onto CA3 pyramidal cells and reduced the peak magnitude of GABA-evoked current in cultured hippocampal neurons via the activation of protein kinases other than protein kinase C [39]. In addition to IL-1β direct postsynaptic effects, numerous in vivo investigations demonstrated that IL-1β modulates exocytosis of a number of neurotransmitters, including adenosine, glutamate, norepinephrine, and GABA in the brain [45]. Specifically, evidence indicates that IL-1β exerts presynaptic effects on glutamatergic terminals, leading to the regulation of glutamate release. For example, intracerebroventricular injection of IL-1β increased levels of glutamate and hydroxyl radicals in organum vasculosum laminae terminalis (OVLT). This IL-1β-evoked rise in OVLT glutamate and hydroxyl radicals could be prevented by intracerebroventricular pretreatment with IL-1Ra [50]. Some reports address the interaction between IL-1β and the endocannabinoid system, based on evidence that the effect of IL-1β on spontaneous excitatory and inhibitory postsynaptic currents in the striatum is regulated by transient receptor potential vanilloid 1 (TRPV1) channels, belonging to the endocannabinoid system. It was shown that the inhibitory action of the cannabinoid CB1 receptor on presynaptic glutamate release was fully blocked in the presence of IL-1β and that the protein kinase C/TRPV1 pathway was involved in this effect [51]. IL-1β can also augment neuronal excitability by elevating extracellular K+ concentrations. The inward rectifying K+ channel 4.1 (Kir 4.1) in astrocytes is crucial for K+ buffering; hence, its inhibition hinders K+ absorption and increases seizure susceptibility. IL-1β reduces Kir 4.1 mRNA and protein levels, potentially contributing to seizures and epileptogenesis [39].

#### 5.1.2. Tumor Necrosis Factor-α

During acute epileptic seizures, the concentration of TNF-α and IL-6 in glial cells also increases, as does the concentration of IL-1β, but their increase is transient [8]. Following SE induced in rats by unilateral 60 min electrical stimulation of the CA3 region of the ventral hippocampus, TNF-α mRNA level reached a peak increase 6 h after seizure onset, then declining to basal levels within 3 days [52]. The available research highlights the complex effects of TNF-α on neuronal excitability and excitotoxicity. The tissue microenvironment has a significant impact on TNF-α properties, including the source of TNF-α release (neuronal vs. glial), the amount of its increase and persistence in tissue, and the relative abundance of its receptor subtypes expressed by targeted cells [53]. Investigating the role of TNF-α and its receptors, TNFR1 and TNFR2, in rats’ susceptibility to seizures induced by intra-amygdala injection of KA or by amygdalar kindling, it was discovered that TNFR1 mediated the seizure activity of TNF-α, while TNFR2 mediated the opposite anticonvulsant effect of this cytokine [54]. Transgenic mice overexpressing TNF-α in astrocytes experience seizures spontaneously, whereas enhanced TNF-α expression in neurons does not cause obvious phenotypic abnormalities, implying that pathologic outcomes are determined by the cytokine’s cellular origin [39]. Extensive literature indicates that TNF-α influences neuronal excitability by modulating glutamatergic neurotransmission in the central nervous system by directly interacting with neuronal receptors or by indirect effects mediated by astrocytes and the microvasculature. This cytokine mostly demonstrated functional interactions with AMPA receptors. TNF-α enhances the synaptic excitatory efficacy by increasing surface expression of AMPA receptors (AMPARs) [55]. Additional study showed that the bulk of glutamate receptor 2 (GluR2)-lacking Ca^2+^-permeable AMPARs are directed to extrasynaptic locations, and their surface levels peak 15 min after TNF-α administration. Although it happens more slowly, TNF-α also causes a rise in GluR2-containing surface AMPARs [56]. TNFα also induced increased surface localization of NMDA receptor NR1 subunits [57]. It has been demonstrated that the subunit composition of glutamate receptors is regulated by intracellular signaling triggered by TNF-α receptor subtypes. The hippocampus levels of NMDA, AMPA, and KA subunits of glutamate receptors were shown to be altered in knockout mice lacking TNFR1 or TNFR2. These modifications aligned with the variations in seizure susceptibility observed in these mice [58]. TNF-α has also been reported to induce the endocytosis of GABA-A receptors, leading to a reduction in surface GABA-A receptors and a diminished inhibitory strength [59]. The inhibition of the glutamate transporter GLUT-1 on astrocytes and the increase of astrocytic glutamate release are two examples of indirect effects of TNF-α on neuronal excitability [53].

#### 5.1.3. Interleukin-6

IL-6 is pivotal in the pathophysiology of inflammatory diseases and in the physiological homeostasis of brain tissue. Neuropathological changes, including multiple sclerosis (MS), Parkinson’s disease, and Alzheimer’s disease, are linked to elevated IL-6 expression in the brain [60]. Alterations in IL-6 expression produced by seizures resemble those documented for TNF. Following unilateral 60 min electrical stimulation of the CA3 region of the ventral hippocampus in rats, the hippocampal cells exhibited an increase in IL-6 mRNA peaking 6 h post seizure onset and lasting several days [52]. IL-6 is predominantly released by activated astrocytes and microglia [61]. Mice with IL-6 overexpression in astrocytes had a decreased threshold for KA-induced seizures and experienced SRSs [62]. IL-6 exerts its actions via binding to either the membrane bound IL-6R (classic signaling) or when it binds to the soluble form of IL-6R (trans-signaling) [63]. Trans-signaling is a primary mechanism for the pathogenic effects of IL-6 in the brain, including inflammatory processes and neurodegeneration, and inhibiting trans-signaling mitigates the adverse effects of IL-6 [60,63]. The molecular mechanisms that have been identified in neuronal cells as IL-6-dependent are linked to a decrease in GABAergic inhibitory activity and an increase in excitatory neurotransmission [39]. IL-6 treatment of cultured rat hippocampal neurons enhanced NMDA-dependent Ca^2+^ fluxes by activating the JAKs/STATs pathway and diminished the levels of AMPA-GluA2 and metabotropic mGluR2/3 receptor subunits, thereby augmenting Ca^2+^ permeability and compromising the inhibitory action on glutamate synaptic release [39].

### 5.2. HMGB1-TLR4 Signaling Pathway

The proconvulsant pathway involving the release of high-mobility group box-1 (HMGB1), a chromatin-binding protein, from neurons and glia and its interaction with Toll-like receptor 4 (TLR4) has been identified using models of acute and chronic seizures in mice [64]. For example, the activated HMGB1 was increased in mice exposed to KA-induced acute seizures [64]. In diazepam (DZP)-refractory and KA-induced SE in mice, the increased HMGB1 rapidly reduced the onset threshold of SE, which was mediated by downstream receptor TLR4 [65]. In epilepsy models, the upregulation of HMGB1 has been seen in the KA-induced chronic epilepsy mouse model and in the rat model of experimental autoimmune encephalitis [66]. HMGB1-associated signaling pathways have been inhibited to evaluate the activity of HMGB1 in various animal seizure and epilepsy models. The inhibition of HMGB1’s action by the broad-spectrum receptor antagonist BoxA or by selective TLR4 antagonists markedly postponed seizure onset and diminished seizure frequency produced by KA or bicuculline in mice [44]. Anti-HMGB1 monoclonal antibody (mAb) attenuated both acute convulsions (maximal electroshock seizure, PTZ-induced convulsions) and chronic seizures (KA-induced) in a dose-dependent manner [67]. An anticonvulsant effect of anti-HMGB1 mAb has also been presented in PILO-induced SE in mice [68]. The anticonvulsant activity of anti-HMGB1 mAb is mediated by downstream TLR4, as TLR4−/− mice exhibited resistance to seizure induction, and the protective effect of anti-HMGB1 mAb is abolished in TLR4−/− mice [67].

In adult rats exposed to SE during epileptogenesis, HMGB1 gradually translocates in the cytoplasm of activated astrocytes and, to a lesser extent, in microglia, neurons, and endothelial cells of the BBB [44]. Following epileptogenic insults, HMGB1 stimulates TLR4 and the receptor for advanced glycation end products (RAGE), which are persistently generated in neurons and activated astrocytes in rodent epileptogenic brain regions [44]. Proconvulsant effects of HMGB1 are mediated by neuronal TLR4 [64] as well as RAGE, which contributes to hyperexcitability underlying acute and chronic seizures and to the proictogenic effects of HMGB1 [69]. The biological effects of HMGB1 are largely determined by the redox state of the extracellular environment: the disulfide (partially oxidized) isoform of HMGB1 activates TLR4 and induces seizures [44].

Similarly to IL-1β signaling, HMGB1-TLR4 pathway affects glutamatergic and GABAergic neurotransmission. The activation of HMGB1 affects glutamate receptor-mediated Ca^2+^ permeability in neurons by increasing NMDA-NR2B or AMPA-GluR2 receptor phosphorylation via Src kinase or PI3-kinase [32]. Reduction of GABA-A receptor-mediated currents and extracellular glutamate increase due to inhibition of astrocytic glutamate re-uptake or its accelerated glial release are other molecular processes that may contribute to HMGB1 hyperexcitability underlying seizures [44].

### 5.3. Cyclooxygenase-2 Signaling

COX-2 plays an important role as a mediator in neuroinflammation. COX-2 activity converts arachidonic acid to prostaglandin G2 (PGG2), which is then reduced by peroxidase to PGH2. PGH2 is quickly converted by particular synthetases into thromboxane A2 (TxA2) and prostaglandins PGF2α, PGE2, PGD2 and prostacyclin I2 (PGI2) [70]. In recent years, there has been a lot of interest in COX-2, an enzyme responsible for the biosynthesis of prostaglandins (PGs), proinflammatory mediators, in experimental seizures. As numerous experimental studies have shown, after SE induction or hippocampal kindling, COX-2 mRNA levels in the adult rodent hippocampus surged quickly and remained elevated for several days [44]. Convulsions generate COX-2 in hippocampal principal neurons within hours partly via a pathway involving NMDA receptors [70]. In a rat hippocampal kindling model, COX-2 induction started in hippocampal neurons and later extended to neocortical neurons. This spreading of COX-2 expression continued when rats were rekindled 34 days later. In this study, nimesulide, a COX-2 specific inhibitor, reduced the development of kindling. On this basis, it was concluded that neuronal COX-2 gene induction is a key signaling event in epileptogenesis [71].

Numerous studies have been conducted to investigate the function of COX-2 both in acute and chronic seizures. However, these studies have produced some controversial and contradictory results. In most acute seizure models, particularly in PTZ convulsions, the inhibition of COX-2 appeared to be neutral or beneficial by increasing the acute seizure threshold [70]. On the other hand, for example, prophylactic prandial administration of rofecoxib, a selective COX-2 inhibitor, lacked efficacy against acute PTZ-induced seizure generation in mice [72]. Pretreatment with nimesulide, the COX-2 inhibitor, augmented KA-induced seizures and increased the mortality rate in rats [73]. Moreover, other COX-2 selective inhibitors (NS-398 or celecoxib) and nonselective inhibitors such as indomethacin aggravated KA-induced seizure activity and the following hippocampal neuronal death [74]. It has been suggested that the observed differences in convulsive activity in response to COX-2 inhibitors’ effects may be at least partially due to the strain/species of the animal, the timing and route of drug administration, the convulsant, and the prostaglandin levels at the time of pharmacological intervention [44,73]. Similar to acute seizures, studies on epileptogenesis have observed discrepancies in the action of COX-2 inhibitors. Pretreatment with nimesulide reduced the development of kindling caused by PTZ [75] or rapid electrical stimulation [76] in mice. In turn, prophylactic treatment with rofecoxib for five days showed no impact on PTZ-kindling in mice [72].

**Figure 2:**
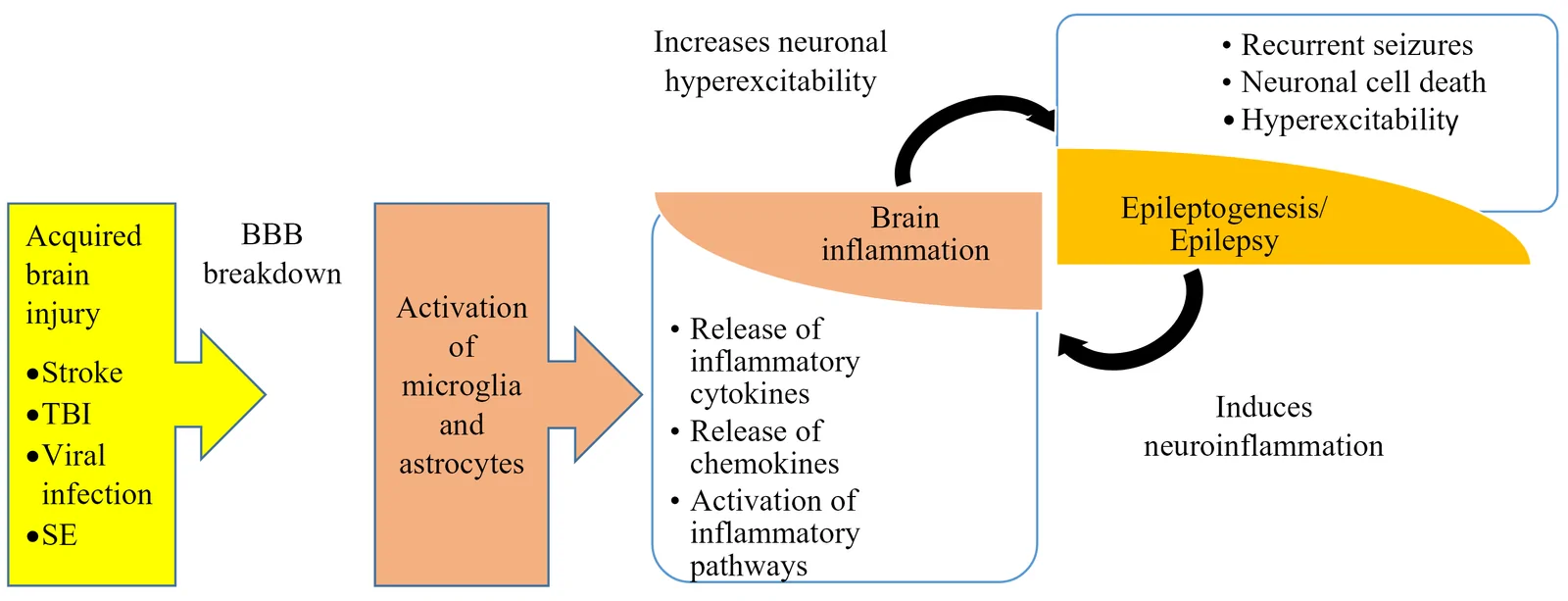
A direct relationship between neuroinflammation and epileptogenesis/epilepsy. Brain inflammation contributes to the development of epilepsy, while seizures induce neuroinflammation.

Even though studies targeting COX-2 suggest that COX-2 inhibition may be beneficial due to the role of this enzyme in epileptogenesis and pharmacotherapy, the adverse cardiovascular and cerebrovascular effects of COX-2 inhibitors severely limit these drugs as therapeutic agents [70]. COX-2 inhibitors have also been examined in terms of the development of SRSs when administered after SE. Following PILO-induced SE, celecoxib treatment for 28 days reduced the number of animals that had SRSs and diminished the frequency and duration of SRSs [77]. In another study, the COX-2 inhibitor parecoxib was neuroprotective but not antiepileptogenic in the PILO model of TLE. Administered after SE, parecoxib prevented the SE-induced increase in PGE2 and reduced neuronal damage in the hippocampus and piriform cortex. Although it reduced the severity of SRSs, it did not alter the incidence, frequency or duration of SRSs developing after SE [78]. Similarly, another COX-2 selective inhibitor, SC-58236, administered following electrically induced SE, did not affect frequency and duration of SRSs [79]. The differences in the efficacy of COX-2 inhibition on SRSs may be attributed to variances in the duration of SE experienced by animals, the dosing regimen of the COX-2 inhibitors, or off-target toxicity [70]. Alternative therapeutic approaches to COX-2 inhibitors may involve the targeting of downstream effector molecules within the COX-2 signaling pathway. Antagonists of the EP1 and EP2 prostaglandin receptors appear to be attractive targets. Particularly, the EP2 receptor is suggested to be involved in the development of neuropathologies following SE [70]. Selective EP2 antagonists, when administered in mice 1 h after termination of PILO-induced SE, reduced neuronal injury in the hippocampus [80]. The EP1 receptor antagonist SC-51089, injected 60 min prior to kindling, decreased the seizure severity in the mouse amygdala kindling model of TLE [81]. The relationship between neuroinflammation and epileptogenesis/epilepsy is shown in Figure 2.

## 6. Oxidative Stress

Oxidative stress is a biochemical condition resulting from the generation of reactive oxygen species (ROS) and reactive nitrogen species (RNS) due to mitochondrial malfunction and increased activity of nicotinamide adenine dinucleotide phosphate (NADPH) oxidase (NOX), xanthine oxidase, and inducible nitric oxide synthase (iNOS) [32]. In the brain, NOX, mitochondria, xanthine oxidase, and lipoxygenase are the principal producers of ROS, while the primary endogenous antioxidants, reduced glutathione (GSH) and α-tocopherol (vitamin E), play crucial protective roles alongside antioxidant enzymes such as superoxide dismutase (SOD), catalase (CAT), glutathione reductase (GR), glutathione peroxidase (GPx), and peroxiredoxins (Prxs) [82]. Oxidative stress, along with glutamate excitotoxicity and neuroinflammation, appear to form a pathogenic “triad” that characterizes the neurobiology of epilepsy, resulting in seizure-induced cell death, increased susceptibility to neuronal synchronization, and network alterations [83]. The production of ROS and inflammation are intricately linked. ROS generation is modulated by inflammatory pathways, and inflammatory processes are influenced by ROS [84]. Through their cytotoxic effects on neurons, disruption of redox-regulated cellular processes, or aggravation of brain inflammation, ROS may contribute to epileptogenesis [84]. Specifically, the potential involvement of oxidative stress in epilepsy and epileptogenesis has been proposed based on the following findings. Research indicated that recurrent seizure activity stimulates the production of ROS, resulting in oxidative damage to cytosolic and mitochondrial proteins, nuclear and mitochondrial DNA, ion channels, neurotransmitter transporters, and hippocampal phospholipids [85]. Additionally, a chronically disrupted GSH redox status and alterations in the activities of antioxidant enzymes (CAT, SOD, GPx) are observed in rodent seizure tests and models of epileptogenesis [82,85]. Furthermore, rodent models and human patients genetically deficient in mitochondrial superoxide dismutase (SOD2) or thioredoxin 2 (TXN2) exhibit SRSs and seizure-related neuropathology [85].

Oxidative stress has been proposed to be a major effect of glutamate excitotoxicity. An epileptogenesis trigger, such as, e.g., SE, increases extracellular glutamate concentrations which are toxic to neurons [82]. Excitotoxicity results in the overactivation of glutamate receptors, NMDA and AMPA receptors, leading to a significant increase in free cytosolic and mitochondrial Ca^2+^ concentrations [86]. The Ca^2+^ entrance and activation of glutamate receptors activate two enzymes: NADPH oxidase (NOX) and nitric oxide synthase (NOS). Further, the formation of ROS and nitric oxide (NO) leads to the production of peroxynitrite, which is harmful to DNA, proteins and lipids. Increased cytosolic Ca^2+^ concentration, resulting in excessive activation of Ca^2+^ signaling pathways, causes mitochondrial malfunction and facilitates the release of cytochrome c into the cytosol, thereby initiating apoptotic pathways. Apoptosis and necrosis resulting from mitochondrial damage induced by Ca^2+^ uptake, mitochondrial Ca^2+^ excess, and ROS formation are processes implicated in epileptogenesis [82].

The role of oxidative stress in epilepsy has also garnered sustained attention due to antioxidant treatment strategies (increasing/supplementing GSH levels and decreasing ROS production) reducing oxidative stress and neuroinflammation and providing neuroprotection [85]. Many antioxidative agents have been evaluated for their capacity to reduce oxidative stress and seizure activity in animal models of epileptogenesis, with not always convincing results regarding their antiepileptogenic properties. Long-term treatment with vitamin E (α-tocopherol) prevented oxidative damage in the hippocampus and increased hilar parvalbumin expression in rats with epilepsy without a reduction in seizure frequency following PILO-induced SE [87]. Although a multitude of animal studies has evidenced the efficacy of different antioxidants, including vitamin C, N-acetyl-cysteine, coenzyme Q10, and various plant extracts or flavonoids, in reducing seizure activity accompanied by diminishing lipid peroxidation and reinstating the functions of various antioxidant enzymes such as SOD, CAT, and GPx, as well as the levels of GSH in diverse brain structures, the translational potential of many of these studies is low, as the antioxidant was given prior to the SE [88]. Currently, there are no data assessing whether vitamin C administration after the occurrence of an epileptogenic insult, such as SE or TBI, reduces the risk of subsequent epilepsy or the number of SRSs. Most available studies examine the prophylactic or very early effect on seizures, rather than the therapeutic effect on epileptogenesis. In other words, the current state of knowledge indicates that vitamin C has documented antioxidant, neuroprotective and anticonvulsant effects in the acute phase, while its antiepileptogenic effect and inhibition of the development of later SRSs remain unproven. Oxidative stress persists for days to weeks after SE and TBI, so the treatment initiated after the injury could still affect the epileptogenic process. The evaluation of vitamin C and other antioxidants on the development of epilepsy is therefore theoretically justified. On the other hand, in PILO- induced convulsions, vitamin C administration before seizure induction significantly prolonged the latency to first seizures, reduced mortality rate, and inhibited LPO in the hippocampus of adult rats [89]. Assuming that SE may be a trigger of epileptogenesis (including PILO-induced SE), the above study might indirectly provide evidence for a possible inhibitory effect of vitamin C on epileptogenesis. However, a study by Kamalimanesh et al. [90] presented a dual effect of vitamin C on PTZ kindling. It was demonstrated that lower doses of vitamin C exert a protective effect against PTZ kindling, while high doses may enhance epileptogenesis.

Additional antioxidant strategies involve reducing ROS production by inhibiting NOX and enhancing intrinsic antioxidant defenses via the activation of nuclear factor erythroid 2-related factor 2 (Nrf2) [88]. Inhibition of ROS production by AEBSF, a NOX inhibitor, markedly reduced seizure-induced cell death in the perforant path model of epilepsy [91]. The transcription factor, Nrf2, is essential for the induction of a battery of phase II detoxification genes through the antioxidant response element (ARE) that lies in their promoter region [92]. It has been established that over 200 genes encoding endogenous protective proteins are regulated by the Nrf2 signaling pathway [93]. They play a crucial role in augmenting tissue oxidative resistance and protecting cells from toxic damage [94].

Although there are some basic studies demonstrating the beneficial effects of common antioxidants, such as, e.g., certain vitamins, on epilepsy, their scope and impact remain limited. Considering the potential introduction of vitamins into epilepsy treatment, this issue must be approached with a balanced perspective. Vitamin supplementation as an adjunctive therapy may be a low-risk and cost-effective strategy for improving outcomes in patients with drug-resistance epilepsy. However, any recommendations should be based on solid evidence from well-designed, large-scale clinical trials [94].

Examples of anticonvulsant/antiepileptogenic activity of different antioxidants and/or anti-inflammatory agents are presented in Table 1.

## 7. Can Antiepileptogenic Drugs Be Found Among Selected Already Registered Medications for Other Disorders than Epilepsy?

### 7.1. Losartan and Candesartan

Among antihypertensive drugs, losartan (blocking angiotensin II type 1 receptors) unexpectedly has been unexpectedly documented to exert antiepileptogenic properties in a model of acquired epilepsy in rats, induced by sodium deoxycholate administered directly to the brain surface, which leads to BBB breakdown, resulting in the exposure of the brain cortex to serum albumin [106]. This procedure results in neuroinflammation with the clear-cut involvement of TGF-β signaling, which leads to epileptiform activity [107]. Losartan (100 mg/kg, i.p.) was initially injected 40 min after sodium deoxycholate and then continued in drinking water (2 g/L) for 3 weeks. The number of rats with SRSs was significantly reduced by losartan—only 40% of animals showed seizure activity, unlike the control group, where all animals exhibited SRSs. Additionally, the average number of convulsions per week was sharply reduced in the losartan group [106].

Losartan has been evaluated in amygdala-kindled rats, evidently extending the development of fully kindled seizures [108], which was reflected in the increased number of electric stimuli necessary to reach the full convulsive response. The drug proved also effective in the post-SE model of epileptogenesis in rats [109]. Losartan was initially given s.c. (10 mg/kg) and continued for the next 3 days following the onset of KA-induced SE. After 3 days, losartan was administered via drinking water for 4 weeks. The occurrence of SRSs was recorded for 3 months. Losartan significantly increased the latency to the onset of SRSs and also provided clear-cut neuroprotection, especially in the CA1 subfield. Losartan-treated rats also performed better in a number of behavioral tests—for instance, diurnal variability in locomotor activity was restored. Some seizure parameters were not, however, affected by losartan—these were seizure severity or afterdischarge duration [109]. It is very likely that the antiepileptogenic activity of this antihypertensive drug is related to the blocking of TGF-β signaling [110].

Candesartan was studied in a knockin mouse model (with a pathogenic Scn8a knockin variant) in both homozygotes and heterozygotes [111]. SRSs start between days 15 and 20 (with survival time of 21–28 days) and in heterozygotes between days 70 and 75 (with much longer survival time up to 150 days). Candesartan either injected subcutaneously at a daily dose of 2 or 4 mg/kg in homo- and heterozygotes or dosed orally in heterozygotes via pellets at a mean daily dose of 9.1 ± 4.2 mg/kg. Evidently, candesartan administration was associated with increased survival and longer seizure freedom. In addition, candesartan reduced the BBB permeability. Importantly, the drug partially normalized profiles in the gene expression throughout the genome in the transgenic mice which resulted in a reduced signaling of NF-κB, TNFα, TGF-β and IL-6 [111].

No clinical trials have been conducted to evaluate the possible antiepileptogenic effects of angiotensin receptor blockers in patients at risk of epilepsy. However, Wen et al. [112], using propensity score matching, analyzed angiotensin receptor blockers (mostly losartan) in comparison with other classes of antihypertensive drugs for their ability to reduce incidence of epilepsy in patients with hypertension. They assessed 619,858 patients to compare angiotensin receptor blockers vs. angiotensin-converting enzyme inhibitors, 619,828 patients for comparing angiotensin receptor blockers vs. beta-blockers, and 601,002 patients to compare angiotensin receptor blockers vs. calcium channel inhibitors. To minimize the confounding effect of stroke, the authors estimated stroke diagnoses with the use of different time intervals—baseline, exposure window, and follow-up period. In their primary analyses, they did not account for stroke occurring during the last two intervals, thus excluding post-stroke epilepsy from the final outcome. The obtained results indicate that in patients taking angiotensin receptor blockers, the incidence of epilepsy was lower than in patients taking other antihypertensive drugs. Moreover, this particular activity of angiotensin receptor blockers may be attributed to their pharmacological profile rather than to a decreased incidence of stroke [112]. It appears that the hypotensive effect of losartan did not play a role in reducing the incidence of epilepsy, as other antihypertensive drugs had a weaker effect in this regard.

### 7.2. Minocycline

A second-generation tetracycline, minocycline, in addition to its pronounced antimicrobial effect, has a potent anti-inflammatory effect [113]. In the central nervous system, this drug inhibits microglial activation and the release of proinflammatory cytokines [114].

Minocycline has been shown to exert some antiepiletogenic activity in rats [115]. Minocycline (45 mg/kg) was given for 2 weeks after the onset of lithium/pilocarpine-induced SE, and SRSs were monitored for 2 weeks after 6 weeks since minocycline discontinuation. Evidently, SRSs were inhibited, which was confirmed by three parameters—their frequency, duration and severity. Apart from suppressed convulsive activity, minocycline pretreatment also led to the reduced activation of microglia and increased production of TNF-α and IL-1β in the hippocampal CA1 subfield and the surrounding cortex. Minocycline pretreatment did not prevent, however, activation of astrocytes [115]. Nevertheless, minocycline was shown ineffective in terms of spontaneous seizure activity in rats following electrically induced SE. Pretreatment with this drug also prevented the development of spatial memory deficit and normalized locomotor activity [116].

Very recently, Postnikova et al. [117] investigated whether minocycline could affect processes related to epileptogenesis in juvenile rats. For this aim, minocycline was administered for seven days (100 mg/kg once daily, immediately after lithium/pilocarpine-induced SE for two days, followed by 50 mg/kg once daily for the next 5 days). It was evident that 7 days after the induction of SE, minocycline treatment resulted in microglial remodeling—a significant increase in the number of lba1-positive microglia in the CA1 and CA3 hippocampal fields was observed. However, minocycline had no effect on microglial cell proliferation in the hippocampus after the onset of SE, which may indicate that minocycline may actually be responsible for modulating the functional state of microglial cells. Microglial remodeling was associated with complete restoration of NMDA receptor-mediated long-term potentiation and after all minocycline rescued currents mediated by NMDA receptors during high-frequency stimulation. Behavioral data obtained 27 days after SE revealed significant reduction in anxiety-like behaviors related to epilepsy, for example, a normalized open field test or reduced self-grooming [117].

### 7.3. Rapamycin and Apigenin

Rapamycin (an immunosuppressive drug) is an effective blocker of the mTOR 1 complex (mammalian target of rapamycin), which, as a serine-threonine kinase, is involved in the synthesis of neuronal proteins [118].

Data on rapamycin are evidently unequivocal. For instance, rapamycin (10 mg/kg) was administered at 10 mg/kg for 2 months after pilocarpine-induced SE in mice [119]. Spontaneous seizure activity was monitored for one month, starting one month after the induction of SE, and mossy fiber sprouting as well as neurodegeneration were assessed 2 months following SE. No effect of rapamycin was found on SRS frequency although mossy fiber sprouting and hypertrophy of the dentate gyrus were reduced. However, no neuroprotection was observed with respect to hilar neurons. When given at 3 mg/kg in mice for 2 months following pilocarpine SE, the drug also remained without any significant effect on spontaneous convulsions and did not prevent the generation of ectopic cells or proliferation of granule cells. Similarly, as in the above study, rapamycin inhibited mossy fiber sprouting and the hypertrophy of the dentate gyrus [120]. In the amygdala stimulation model of rat TLE, the animals were given rapamycin at 6 mg/kg daily for 2 weeks, and SRS activity was monitored during the drug administration and during the following 4 weeks [121]. No significant effects were found in terms of the number of rats developing SRSs, latency to SRS activity or SRS frequency. Also, mossy fiber sprouting was not affected by rapamycin [121]. Shima et al. [122] induced SE by intrahippocampal KA in mice and then, 5 h after, administered rapamycin (80 mg/kg) and subsequently continued rapamycin at 40 mg/kg daily for 20 days. Although rapamycin inhibited granule cell dispersion and mossy fiber sprouting, it did not affect hippocampal paroxysmal discharges evaluated 25 days after intrahippocampal KA [122].

In rats, following SE induced by the electrical stimulation of the angular bundle, rapamycin (6 mg/kg) was given to rats for 3 weeks, and the animals were monitored for the next 5 weeks [123]. Interestingly, SRS activity was suppressed within the first 3 weeks and then continued to re-appear when rapamycin administration was stopped. In this study, rapamycin (3 mg/kg) was also injected 3 days prior to the induction of SE or during the phase of SRSs. In the first case, the development of SRSs was not prevented, whilst 5-day rapamycin administration in the chronic phase led to the reduction of SRS frequency [123].

Nevertheless, rapamycin seemed to affect SRS activity, as reported by some authors. Van Vliet et al. [124] used the electrical stimulation of the rat angular bundle as a method to induce SE. Rapamycin, following SE, was given at 6 mg/kg daily for a week and then continued once every two days for six weeks. SRS activity in rapamycin-treated rats was either stopped in 25% of the animals or significantly reduced in the remaining 75%. Further, rapamycin pretreatment was clearly associated with a distinct neuroprotective effect in the hippocampal area. Also, the increased permeability of the blood–brain area was reduced in these rats [124]. Guo et al. [125] evaluated rapamycin in a mouse model of TBI. Rapamycin was given at 6 mg/kg one hour after brain injury and its administration was continued once daily for up to 4 weeks. Following the controlled cortical impact, the animals were monitored continuously with a video–EEG system for the occurrence of electrographic seizures for 16 weeks. In only 12.5% (2 of 8) rapamycin treated mice, spontaneous electrographic seizures were present, compared to the vehicle-treated group, in which 50% of mice developed PST. Moreover, the control animals exhibited almost 10 times greater seizure frequency than the rapamycin-treated mice. Rapamycin also inhibited neurodegeneration and mossy fiber sprouting, but interestingly, the sprouting was reversed after rapamycin had been stopped [125].

Apigenin (not clinically approved), a plant-derived m-TOR antagonist [126], was evaluated in rats after intracerebroventricular KA-induced SE. The drug was administered orally at a daily dose of 50 mg/kg for 6 days, starting from 5 days before the induction of SE. The number of spontaneous seizure spikes, evaluated for 30 min by intracranial EEG recording one month after SE, was significantly reduced in apigenin-pretreated rats. Moreover, spontaneous seizure spikes, neurodegeneration, aberrant neurogenesis and mossy fiber sprouting and m-TOR hyperactivity were also decreased by apigenin pretreatment at 50 mg/kg for six days, following intracerebrovetricular KA-induced SE in rats [127].

### 7.4. Statins

Statins are effective inhibitors of 3-hydroxy-3-methylglutaryl-coenzyme A reductase, and the mechanism of action of this drug is to reduce cholesterol biosynthesis and, consequently, reduce the cholesterol fraction in plasma and in the brain [128,129]. In addition, they also exert anti-inflammatory, anti-oxidative, and neuroprotective effects [30,128,130], which makes this class of drugs effective in a number of neurological conditions. Statins have been reported to protect against seizure activity in different animal seizure models [131] and to potentiate the anticonvulsant activity of some antiseizure medications [132,133]. That is why all statins were considered as potential antiepileptogenic drugs and the available experimental and clinical evidence is encouraging [134].

Atorvastatin seems the most frequently evaluated statin in terms of antiepileptogenesis. Oliveira et al. [135] intragastrically administered atorvastatin (10 or 100 mg/kg) for 14 days to mice surviving pilocarpine-induced SE. Then, on days 7 and 14, the susceptibility of mice to PTZ (at a low dose of 30 mg/kg) was evaluated. It was evident that latencies to myoclonic jerks or tonic–clonic seizures were shortened as compared to baseline (prior to SE), which may point to the development of enhanced seizure susceptibility. No neuroprotective activity of atorvastatin was found in the hilus of the dentate gyrus. In the same experimental approach [135], atorvastatin-treated mice showed better performance in the open field and object recognition tests. The statin also dose-dependently reduced concentrations of basal and SE-elevated pro-inflammatory factors in the cortex and hippocampus. In contrast, the level of anti-inflammatory IL-10 was increased by atorvastatin [136]. In addition, lovastatin exerted a similar effect on the concentration of hippocampal inflammatory mediators and IL-10 in the chronic phase after pilocarpine-induced SE in rats [137]. A number of statins (atorvastatin 5 and 10 mg/kg/day, simvastatin 10 mg/kg/day, pravastatin 10 and 30 mg/kg/day, all administered orally) were tested for 17 consecutive weeks for the appearance of absence seizures (verified by EEG) in WAG/Rij rats, serving as a genetic model of absence epilepsy [138]. The statins were also given acutely in rats with already developed absence seizures. Evidently, chronic administration of statins (atorvastatin and simvastatin at 10 mg/kg/day, pravastatin at 30 mg/kg) in 45-day-old rats reduced the development of absence seizures in rats at 6 months of age. The last dose of statins was administered one month before evaluation of seizure activity. Interestingly, reduced seizure activity was also observed in rats 5 months after discontinuation of statin administration. On the other hand, acute administration of statins to 6-month-old rats did not produce any anticonvulsant effects [138].

Van Vliet et al. [139] treated rats orally with atorvastatin (10 mg/kg/daily) for 14 days. The treatment was started 7 days prior to SE evoked by electrical stimulation of the angular bundle. The development of SRS activity was not affected by atorvastatin, and, moreover, BBB leakage was not prevented. In the end, atorvastin did not modify hilar cell loss and mossy fiber sprouting [139].

Meta-analysis studies are available that consider the antiepileptogenic effects of statins in clinical settings. Acton et al. [140] identified 182 citations related to post-stroke epilepsy, of which 175 were excluded due to ineligibility or duplication. The remaining publications, in the form of cohort studies, included a total of 53,579 patients. Post-stroke use of statins resulted in reduced risk of post-stroke epilepsy and early onset seizures as well. Another meta-analysis by Fang et al. [141] encompassed seven studies including 40,381 patients. They also concluded that statins reduced the risk of post-stroke epilepsy and especially that of early post-stroke seizures.

An interesting question arises as to whether the anticonvulsant and potentially antiepileptogenic effects of statins might also be linked to a reduction in brain cholesterol levels. This possibility seems plausible, even though cholesterol does not cross the blood–brain barrier [142]. Cholesterol synthesis and metabolism occur within the brain, with cholesterol 24-hydroxylase serving as the primary metabolizing enzyme. The resulting 24S-hydroxycholesterol acts as a positive modulator of NMDA receptors and can enhance glutamate release via neuronal pathways dependent on TNF-α. Moreover, increased stimulation of NMDA receptors leads to an increase in cerebral cholesterol metabolism, ultimately creating a vicious circle causing brain hyperexcitability. Interestingly, inhibition of cholesterol 24-hydroxylase exhibits anticonvulsant effects and increases survival in animal models of epilepsy [142]. Thus, the antiepileptogenic action of statins could be linked to their influence on brain cholesterol metabolism, in addition to their pleiotropic effects, related to neuroprotection and inhibition of inflammation, oxidative stress and protection of blood–brain barrier function [30,128,130,143].

The antiepileptogenic potential of drugs registered for other disorders than epilepsy are shown in Table 2.

## 8. Antiepileptogenic Potential of Selected Newer Antiseizure Medications

### 8.1. Levetiracetam and Brivaracetam

Both levetiracetam and brivaracetam exhibit high affinity for the synaptic vesicle protein SV2A, with brivaracetam binding 20 times more strongly. There is an obvious correlation between the binding affinity for the SV2A site and the degree of anticonvulsant activity [144].

Levetiracetam (administered intracerebroventricularly via osmotic minipumps for 25 days following KA-induced SE) effectively reduced the number of spontaneous seizures in rats and reduced aberrant neuroogenesis in the hippocampus [145]. Levetiracetam was also administered orally to mice at doses of 250 and 500 mg/kg twice daily for 28 days following pilocarpine-induced SE [146]. Evidently, levetiracetam (500 mg/kg) significantly reduced the incidence of spontaneous recurrent seizure activity within 28 days. Moreover, the drug decreased SE-induced mortality and brain edema and prevented the loss of BBB integrity [146]. A very interesting approach was presented by Silva et al. [147], who administered levetiracetam and brivaracetam to Tg2576mice used as a model of Alzheimer’s disease. Aged Tg2576 mice (13–25 months old) were administered either brivaracetam (10 mg/kg daily) or levetiracetam (150 mg/kg daily) subcutaneously via osmotic minipumps for 28 days; following a one-week washout period after the cessation of drug treatment, amygdala kindling was initiated. Both levetiracetam and brivaracetam significantly delayed the development of kindling compared to the vehicle-treated group. Brivaracetam (10 mg/kg daily) was also given to young Tg2576mice (4–6 months) for 28 days. Then, after a week, the procedure for inducing kindling was initiated. The acquisition of kindling was again delayed by the prior administration of brivaracetam [147].

A series of positive results regarding levetiracetam and brivaracetam were obtained in a rat model of post-traumatic seizures. Following controlled cortical impact, the rats were immediately administered levetiracetam (60–150 mg/kg, i.p.) [148]. Two to three weeks after the injury, cortical slices were examined ex vivo for epileptiform activity. It was found that the administration of levetiracetam increased the intensity of the stimulus required to induce epileptiform activity by a factor of 2 to 4. The percentage of rats and cortical slices exhibiting spontaneous epileptiform bursts was also reduced by the administration of levetiracetam [148]. In the same model of cortical neurotrauma in rats, Ling et al. [149] evaluated brivaracetam (21 or 100 mg/kg, i.p.), administered immediately (0–2 min) and 30 and 60 min post injury. The percentage of evoked and spontaneous bursts (in rats and slices) as well as burst threshold and duration (only in slices) were studied. Brivaracetam (21 mg/kg, administered immediately after the injury) effectively influenced all the above-mentioned parameters, demonstrating its antiepileptogenic effect. When given 30 min after the injury, only burst duration remained unchanged. At 100 mg/kg, brivaracetam exhibited potent antiepileptogenic activity, with only evoked bursts in rats being not significantly affected, when the drug was administered 60 min post trauma [149]. Finally, Mejia-Bautista et al. [150] demonstrated that early administration of brivaracetam (21 mg/kg) following cortical injury inhibited the rats’ increased sensitivity to the convulsant effects of a low dose of 4-aminopyridine.

However, in the case of SE induced in rats by pilocarpine, lithium/pilocarpine, or stimulation of the basal amygdala, followed by spontaneous seizure activity, no antiepileptogenic effect of levetiracetam was observed [for review, [151]]. Following pilocarpine-induced SE, levetiracetam was administered as a single intraperitoneal dose of 54 mg/kg, followed by continuous administration via osmotic minipumps at doses of 50, 150, and 300 mg/kg for three weeks. In rats subjected to lithium/pilocarpine-induced SE, the drug (150 mg/kg) was administered i.p. for 5 days. Finally, following stimulation of the basal amygdala, the animals received levetiracetam via osmotic minipumps for 5 or 8 weeks. The infusion parameters were selected to ensure a maximum plasma concentration of levetiracetam of approximately 40 µg/mL [for review, [151]]. Some more recent data also indicate a lack of antiepileptogenic activity of levetiracetam. Kantarci et al. [152] administered the drug at a daily dose of 100 mg/kg to pregnant female Genetic Absence Epilepsy Rats from Strasbourg. The studies were conducted on adult offspring that develop synchronized spike-and-wave discharges. There was no effect of levetiracetam on this characteristic EEG recording [152]. As demonstrated by Kumar et al. [153] in rats following lithium/pilocarpine-induced SE (lasting 90 min), the administration of levetiracetam (400 mg/kg i.p.), given 15 min after the induction of SE and again 4 h later, did not significantly affect the onset or frequency of spontaneous seizures.

### 8.2. Gabapentin

The mechanism of action of gabapentin involves inhibition of the α2δ1 subunit of the voltage-gated calcium channel, resulting in the inhibition of calcium ion influx into the neuron and a reduction in its excitability [144].

Following KA-induced SE, the drug was administered at 200 mg/kg i.p. to immature rats twice a day for one month and then continued at 100 mg/kg (twice daily for 10 days). The incidence of spontaneous seizures was significantly reduced by gabapentin and modest neuroprotection in the hippocampus was observed. Furthermore, no deficits in learning in the water maze test were found as compared to the vehicle group, and increased activity in the open field test was normalized [for review, [151]]. Gabapentin (100 mg/kg, i.p. daily, 3 times daily, 3 h after SE) was also studied in a focal neocortical SE model in mice [154]. In this model, diverse pathophysiological mechanisms lead to epileptogenic activity. Evidently, gabapentin reduced seizure-induced excitatory synaptogenesis as well as exerted neuroprotective effects. In the lithium/pilocarpine model, gabapentin was given at 400 mg/kg/day to rats for 4 days, starting one day after SE [155]. After 21 days, the animals received another dose of lithium chloride and 20 h later were re-injected with pilocarpine at a subconvulsive dose of 10 mg/kg repeated every 30 min. A maximum of 4 doses of pilocarpine were administered. Gabapentin pretreatment effectively reduced the severity of seizures, rats’ mortality and the number of animals with SE. Moreover, gabapentin diminished blood-borne cell infiltration into the brain and reduced microglial cell reactivity [155].

### 8.3. Topiramate

Topiramate has a number of mechanisms of action manifested through the enhancement of GABA-mediated inhibition, modulation of voltage-gated sodium channels, inhibition of carbonic anhydrase, and, likely, modulation of voltage- and receptor-gated calcium channels [143].

In the rat lithium/pilocarpine SE, topiramate was administered at 10, 30, and 60 mg/kg (1 and 10 h after the onset of SE), followed by twice-daily administration for the next 6 days. Across the entire dose range, topiramate exerted a neuroprotective effect against SE-induced damage in the hippocampus, but without affecting the latency or frequency of spontaneous seizures. In another study using the same model, topiramate (10–60 mg/kg) was administered for one week. As before, a neuroprotective effect was observed, with no effect on spontaneous seizures [for review, [151]]. A more recent study [156] utilized pilocarpine-induced status epilepticus (SE) in rats. Administration of topiramate (80 mg/kg, p.o.) was initiated 3 h after SE and continued for 12 weeks. Spontaneous seizures were recorded over a 24 h period after 8 weeks. In this experimental setup, the frequency of spontaneous seizures was significantly inhibited. Interestingly, no effect of topiramate on increased oxidative stress was observed. No effect of this drug on the concentration of pro-inflammatory markers in the hippocampus was observed either [156].

Conclusions drawn from the analysis of the antiepileptogeic potential of antiseizure medications indicate that their mechanisms of action may represent only one of the key factors determining the potential inhibition of epileptogenesis. Other significant factors, such as their dosage, timing, and duration of use, can be equally important. When spontaneous seizures are assessed during continuous drug administration, the inhibitory effect on the process of epileptogenesis may result from an anticonvulsant action, an antiepileptogenic action, or both. It seems that these principles apply not only to antiseizure medications.

## 9. Combined Treatment as an Efficient Way to Halt Epileptogenesis?

This assumption may be based on the results obtained by Welzel et al. [157,158]. In one of the first attempts to identify such an effective drug combination, Welzel et al. [157] selected 10 drugs (including three antiseizure medications, levetiracetam, gabapentin, topiramate, ceftriaxone, celecoxib, and atorvastatin) representing different pharmacological mechanisms thought to be important in epileptogenesis. Combinations of 2–4 drugs were given for three days 16–18 h after intrahippocampal KA-induced SE in mice. Although the majority of combinations were well tolerated, in no case was neuroprotection observed. In their next attempt, Welzel et al. [158] studied 14 drugs sharing various mechanisms proved to affect the process of epileptogenesis. Combinations of 2–4 drugs were given to mice for five days, 6 h after KA-induced SE. Spontaneous electrographic or electroclinical seizures were monitored for seven days, starting 4 and 12 weeks after SE. The most pronounced effect in preventing SRS activity was demonstrated with the combination of levetiracetam, atorvastatin, and ceftriaxone, which reduced the incidence of electrographic seizures by 60% and electroclinical seizures by 100% at 12 weeks. Strikingly, this preventive effect was obtained at low doses of combined drugs and was not observed at higher doses. Interestingly, none of the combinations that were studied exerted neuroprotective effects in the hippocampus damaged by KA [158]. Certainly, levetiracetam and ceftriaxone also have some antiepielptogenic potential when given alone [30,159]. Apart from this very effective combination, Welzel et al. [158] identified some other combinations of drugs targeting various mechanisms of action and providing significant inhibition of SRS activity. Results of this study clearly indicate that effective inhibition of epileptogenesis can be achieved by using a combination of drugs. Moreover, comparison of the results of both studies above [157,158] also indicates the time of administration and the duration of use of the drug combination, which may determine their activity or lack of preventive effect. Another important fact cannot be overlooked—it is particularly important that the pronounced antiepileptogenic effect was associated with low doses of combined drugs, which may be of key importance when planning future clinical trials. Levetiracetam is a modulator of synaptic vesicle 2A, and this target seems to be responsible for levetiracetam’s antiseizure and antiepieleptogenic effects [30]. Ceftriaxone (a β-lactam antibiotic) has been shown to reduce epileptogenesis, primarily by increasing glutamate clearance after brain injury, which apparently reduces glutamate neurotoxicity [30]. Atorvastatin’s anti-inflammatory, antioxidative, and neuroprotective actions were mentioned above. Therefore, these mechanisms are very likely to be responsible for the final antiepileptogenic effect. Notably, some combinations with losartan were also effective [158]. Perhaps further research will also uncover effective combinations using the other mechanisms (targets) presented above.

Another example of efficient combination treatment, shown in Table 1, involves the antioxidants N-acetylcysteine and sulforaphane, which increase brain glutathione levels through a complementary mechanism [104]. It turned out that, in rats, SE induces oxidative stress in both neurons and astrocytes during ongoing epileptogenesis. N-acetylcysteine and sulforaphane were administered in combination during the two-week period of epileptogenesis, and this regimen inhibited oxidative stress much more strongly than the use of either antioxidant alone. This combination significantly prolonged the latency to the onset of seizures and inhibited disease progression during the 2–5-month period following the induction of SE. Furthermore, the frequency of spontaneous seizures assessed after 5 months was markedly reduced. However, the average seizure duration was not affected. Nevertheless, other beneficial effects were noted, as decreased neuronal loss in the hippocampus and reduced cognitive impairments. It is possible that the aforementioned effects of the combination used may result from the inhibition of HMGB1 production induced by oxidative stress [104].

## 10. Conclusions

As noted above, epileptogenesis involves numerous signaling pathways and pathological processes. Therefore, it is reasonable to assume that targeting any one of the numerous mechanisms leading to epileptogenesis and subsequent seizures and comorbidities (such as cognitive decline) may not be sufficient to inhibit epileptogenesis with a disease-modifying effect.

The combination of levetiracetam, ceftriaxone, and atorvastatin, characterized above by its diverse mechanisms of action and significant antiepileptogenic effect [158], serves as an excellent example confirming the rationale for using drug combinations. This assumption is further reinforced by the fact that the use of the aforementioned drugs individually produced no significant—or only a weak—antiepileptogenic effect.

Drug combinations may have synergistic, additive or antagonistic effects, which can be determined by isobolographic analysis, also in the case of triple drug combinations with respect to anticonvulsant activity [160]. A large number of two-antiseizure-drug combinations have been characterized using this method. This has enabled the detection of anticonvulsant combinations with synergistic anticonvulsant activity and antagonistic adverse effects, which are clearly the best from a preclinical perspective [161]. Such combinations included the following: lamotrigine + valproate as well as topiramate + lamotrigine. On the other hand, a negative combination of lamotrigine + oxcarbazepine exerted anticonvulsant antagonism and adverse synergy. The isobolographic method could therefore be used in further searches for combinations of antiepileptogenic drugs, which would allow for their precise characterization in terms of synergistic interactions and the grade of adverse effects. Positive preclinical data could point to the use of appropriate drug combinations in patients at risk of epileptogenesis.

Clinical trials aimed at finding effective preventive strategies with antiepileptogenic properties would undoubtedly encounter significant difficulties. As Engel et al. [162] pointed out some time ago, testing antiepileptogenic methods would involve enormous financial outlays, mainly due to the time required for epilepsy to develop (circa 10 or more years), even after severe epileptogenic insults. It is expected that any future antiepileptogenic clinical trials will identify a patient population that is most likely to develop epilepsy following epileptogenic insults. For example, recurrent seizures were observed in 47 (2.5%) of 1897 stroke patients, and late seizures (occurring no earlier than 2 weeks after stroke) were a clear risk factor for recurrence. This did not apply to patients with hemorrhagic stroke [163]. Regarding posttraumatic epileptic seizures, the risk of their occurrence, following very severe injuries, reached 7.1% within one year and 11.5% over 5 years [164]. These data openly and strongly indicate that for financial and ethical reasons only a small proportion of patients with potential epileptogenic insults may be included in clinical trials. It is therefore obvious that reliable markers for detecting epileptogenic processes are necessary to reduce the number of patients with brain injuries who are likely to experience recurrent epileptic seizures. Different types of such markers are beginning to appear in patients with stroke (ischemic and hemorrhagic) and in patients with TBI. Wang et al. [165] suggest the prognostic significance of low GABA levels in the cerebrospinal fluid and low levels of neuron-specific enolase and micro-RNA-155 in the serum of 69 patients with post-ischemic stroke epilepsy as compared with patients with non-post-stroke epilepsy. In another study [166], the significance of the neutrophil-to-lymphocyte (NLR), platelet-to-lymphocyte, and hemoglobin-to-lymphocyte ratios for the development of post-stroke epilepsy was assessed in 1445 patients with ischemic stroke. Seizures occurred in 43 patients (2.98%) over a median follow-up of 7 years. It was found that NLR was significantly increased in patients with epileptic seizures compared to post-stroke patients without epileptic seizures [166]. In relation to primary intracerebral hemorrhage [167], the relationship between the monocyte-to-lymphocyte ratio (MLR) was assessed for its significance in predicting post-stroke epilepsy. Of 834 patients, 47 (5.6%) developed post-stroke epilepsy. Taking into account several confounders, using multivariate logistic and linear regression analysis, increased MLR levels were found to be associated with the occurrence of post-stroke epilepsy. A new MNCAVE scale has been proposed to assess the risk of post-stroke epilepsy, which, in addition to the MLR, takes into account additional risk factors, i.e., severe stroke, cortical location, young age, large hematoma volume, and the occurrence of early seizures [167]. It has also been suggested that inflammatory markers in blood may have predictive value for the occurrence of epilepsy in patients with TBI [168]. The study encompassed 138 patients with PST and 150 patients with TBI. In patients with epilepsy, NLR and neutrophil count were elevated compared to patients after brain trauma without epilepsy [168]. Inflammatory markers were also assessed in patients with brain injury who were predicted to be at significant risk of developing PST [169]. Blood samples were taken on days 2 and 4 after brain trauma and all the patients were followed for 24 months for the occurrence of epilepsy. Generally, the authors are of the opinion that blood inflammatory markers measured at 2 and 4 days after brain injury may not serve as reliable predictors of PST. However, they observed a significantly faster decline in IL-6 levels in patients with non-seizure-related brain injury (*N* = 73) compared to patients with PST (*N* = 13) [169].

Given that some studies on epileptogenesis markers had low patient numbers, more studies of this type may provide answers regarding the most reliable markers. It is likely that in the near future, recommendations for patient enrollment in studies of effective antiepileptogenic therapies will be developed based on markers with high predictive value.

It appears that dietary therapies could represent a new approach to preventing epilptogenesis. For example, the ketogenic diet not only demonstrates an anti-seizure effect but also positively influences comorbidities—specifically cognitive and psychiatric issues—thereby exhibiting disease-modifying activity and a partial antiepileptogenic effect. The mechanism responsible for these effects could be an increase in cerebral adenosine concentration and the associated inhibition of DNA methylation [170]. An innovative ketogenic diet was administered to rats in a rapid kindling model of epileptogenesis with very good results [171]. Also, other diets were considered—for example, a low-glycemic-index diet in synapsin II knockout mice, which develop seizure activity 2–3 months after birth. Interestingly, epileptogenesis was inhibited in a gender-specific fashion, because only female mice exhibited elevated cortico-hippocampal allopregnanolone concentrations [172].

Undoubtedly, drugs and agents demonstrating antiepileptogenic effects in animal models may exhibit similar potential in clinical studies. They can also serve as a reference point for further research. However, as noted by Klein et al. [173], preclinical research is flourishing, yet clinical impact is lacking. According to these authors, there is a need for new proof of concept and reliable biomarkers to conduct clinical trials on the inhibition of epileptogenesis.

## Figures and Tables

**Table 1:** Effects of selected agents/drugs with anti-inflammatory and/or antioxidant properties in animal models of epileptogenesis.

Drug	Anti-Inflammatory/Antioxidant Effects in the Brain Tissue	Model of Epileptogenesis	Anticonvulsant Effect	References
human recombinant IL-1Ra	IL-1 receptor antagonist; blockade the binding of IL-1β	electrical kindling in rats + LPS	inhibited kindling progression	[95]
VX-765	IL-1β converting enzyme/caspase-1 inhibitor; blockade of IL-1β synthesis	electrical kindling in rats	blocked the development of kindling	[96]
VX-765 + CyP	IL-1β converting enzyme/caspase-1 inhibitor + TLR 4 antagonist; blockade of IL-1β synthesis	intra-amygdala KA model in mice	inhibited the SRS progression, reduced the number of seizures	[97]
synthetic miR-146a	negative feedback regulator of inflammation; suppression of NF-κB signaling	intra-amygdala KA model in mice	inhibited the SRS progression, reduced the number of seizures	[97]
anti-HMGB1 mAb	Anti-HMGB1 monoclonal antibody; inhibition of HMGB1 activity	KA model in mice	reduced the seizure frequency	[67]
anti-HMGB1 mAb	Anti-HMGB1 monoclonal antibody; inhibition of HMGB1 activity	electrical kindling in mice	decreased the seizure severity	[67]
Tocilizumab	humanized monoclonal antibody against the IL-6 receptor; inhibition IL-6 signaling	WAG/Rij rats	reduced the development of absence seizures	[61]
Nimesulide	COX-2 inhibitor; inhibition of prostaglandin production	electrical kindling in rats	attenuated kindling development	[71]
Celecoxib	COX-2 inhibitor; inhibition of prostaglandin production	PILO model in rats	diminished the frequency and duration of SRS	[77]
SC-58236	COX-2 inhibitor; inhibition of prostaglandin production	electrical SE model in rats	no antiepileptogenic effects	[79]
Resveratrol	non-flavonoid polyphenol; reduced the levels of IL-1β, IL1-Ra, IL-6, and TNF-α	PTZ kindling in mice	suppressed the development of kindling	[98]
Hesperidin	flavonoid; attenuated alterations in LPO, GSH, nitrate, SOD and CAT levels, as well as mitochondrial complex (I, II, and IV) activities	PTZ kindling in mice	decreased the seizure score	[99]
N-acetylcysteine	precursor of GSH; inhibited the LPO level	KA model in rats	prevented the neuronal cell loss, mossy fiber sprouting, and increased the threshold for seizures induced by flurothyl ether	[100]
Coenzyme Q10	endogenous antioxidant; decreased the LPO and nitrite concentration, restored SOD, CAT, GSH levels and activities of mitochondrial enzyme complex (I, II and IV)	PTZ kindling in mice	reduced the kindling score	[101]
Lipoic acid	endogenous antioxidant; reduced the level of MDA and NO, increased SOD and CAT activities, promoted the translocation of Nrf2 in the nuclear fraction	PTZ kindling in rats	reduced the total frequency of seizures	[93]
Curcumin	polyphenol found in turmeric; decreased the level of MDA, increased GSH level	PTZ kindling in rats	increased the latency to myoclonic jerks, clonic and generalized tonic–clonic seizures, improved the seizure score	[102]
Dimethyl fumarate	activator of Nrf2; increased the levels of SOD, GPx and GSH; reduced the LPO	PTZ kindling in rats	decreased the number of kindled animals	[103]
N-acetylcysteine + Sulforaphane	precursor of GSH + activator of Nrf2; increased the GSH level, reduced GSSG, GSSG/GSH ratio and GS-Pro, prevented HMGB1 generation	electrical SE model in rats	delayed the onset of SRS, blocked the SRS progression, caused 70% SRS reduction	[104]
RTA 408	activator of Nrf2; increased the total GSH level	KA model in rats	reduced (by 94%) the frequency of SRS	[105]
Ascorbic acid	vitamin C; exogenous antioxidant	PTZ kindling in rats	low doses prevent the progression of seizures; high doses exacerbate seizures	[90]

Anti-HMGB1 mAb, anti-HMGB1 monoclonal antibody; CAT, catalase; COX-2, cyclooxygenase-2; CyP, cyanobacterial product—selective antagonist of bacterial LPS; GPx, glutathione peroxidase; GSH, reduced glutathione; GS-Pro, glutathionylated protein; GSSG, glutathione disulfide; HMGB1, high-mobility group box-1; IL-1Ra, Interleukin-1 receptor antagonist; KA, kainate; LPO, lipid peroxidation; LPS, lipopolysaccharide; MDA, malondialdehyde; NF-κB, Nuclear factor kappa-light-chain-enhancer of activated B cells; NO, nitric oxide; Nrf2, nuclear erythroid-2-related factor 2; Pilo, pilocarpine; PTZ, pentylenetetrazol; SE, status epilepticus; SOD, supeoxide dismutase; SRS, spontaneous recurrent seizure; TLR4, Toll-like receptor 4; TNF-α, tumor necrosis factor-α.

**Table 2:** Antiepileptogenic potential of drugs registered for the treatment of disorders other than epilepsy.

Drugs		Treatment		Experimental Model	Effect	References
		Dose	Drug Exposure Time			
Losartan		100 mg/kg/daily, i.p.	administered 40 min after sodium deoxycholate, followed by losartan 2 g/L in drinking water for 3 weeks	rat model of acquired epilepsy induced by sodium deoxycholate causing BBB breakdown	no seizure activity in 60% of the rats, markedly decreased weekly seizure frequency.	[106]
		50 mg/kg/daily, i.p. for 21 consecutive days	chronic administration during kindling development	amygdala-kindled rat model	increased number of electrical stimulations, required to induce generalized convulsions.	[108]
		10 mg/kg/daily, s.c.	initially and for 3 days after status epilepticus, then oral administration in drinking water for 4 weeks	post-status epilepticus rat model induced by KA	significantly increased latency to spontaneous seizures, neuroprotective effect, particularly in the hippocampal CA1 region, improved behavioral outcomes—restoration of circadian locomotor variability, no changes were observed in the severity of seizures or the duration of the post-seizure state	[109]
Candesartan		subcutaneously at a daily dose of 2 or 4 mg/kg in homo- and heterozygotes or oral pellet administration (mean 9.1 ± 4.2 mg/kg/daily) in heterozygotes	they were given the medicine every day until their death	scn8a pathogenic knockin mouse model (homozygous and heterozygous)	prolonged survival, increased seizure-free period, reduced BBB permeability reduced activity of the NF-κB, TNF-α, TGF-β and IL-6 signaling pathways, which was caused by the partial normalization of gene expression profiles across the entire genome in transgenic mice.	[111]
Minocycline		45 mg/kg/daily	treatment for 2 weeks after status epilepticus	lithium/pilocarpine-induced status epilepticus in rats	significant reduction in spontaneous seizure frequency, duration, and severity, reduced activation of microglia, reduction of TNF-α and IL-1β production in hippocampal CA1 and surrounding cortex	[115]
		0–1 day: 2 × 50 mg/kg, i.p. 2–13 day: 25 mg/kg/daily, i.p.	at subchronic doses, administered immediately following an electrically induced status epilepticus in rats	electrically induced status epilepticus in rats	no significant effect on the occurrence of spontaneous seizures, prevention of spatial memory deficits and normalization of locomotor activity—reduction in hyperactivity and excessive physical activity	[116]
		100 mg/kg/daily 50 mg/kg/daily	100 mg/kg daily for 2 days after status epilepticus, followed by 50 mg/kg daily for 5 days	juvenile rat model of lithium/pilocarpine-induced status epilepticus	significant increase in the number of lba1-positive microglia in the CA1 and CA3 hippocampal fields, reduction of anxiety-like behavior—normalization of open-field activity and self-grooming, restoration of NMDA receptor-mediated long-term potentiation	[117]
Rapamycin		10 mg/kg/daily	for 2 months after status epilepticus	pilocarpine-induced status epilepticus in mice	no reduction in spontaneous seizure frequency despite reduced mossy fiber sprouting and dentate gyrus hypertrophy, no neuroprotection was observed with respect to hilar neurons, no neuroprotection was observed with respect to hilar neurons	[119]
		3 mg/kg/daily	for 2 months following status epilepticus	pilocarpine-induced epilepsy in mice	no significant effect on spontaneous seizures, did not prevent the formation of ectopic cells or the proliferation of granular cells, inhibited mossy fiber sprouting and the hypertrophy of the dentate gyrus	[120]
		1.5 or 3 mg/kg/daily	treatment was started 24 h after the end of pilocarpine treatment and continued for 2 months	pilocarpine-induced epilepsy in mice	no significant effect on spontaneous seizure frequency, no effect proliferation of granule cells, neurodegeneration in the hippocampal hilus or generation of ectopic granule cells, chronic treatment inhibited mossy fiber sprouting and hypertrophy of the dentate gyrus	[120]
		6 mg/kg/daily	for 2 weeks	amygdala stimulation model of temporal lobe epilepsy in rats	no effect on seizure onset, latency, seizure frequency, or mossy fiber sprouting	[121]
		80 mg/kg 40 mg/kg/daily	the first dose, administered 5 h after a status epilepticus continuation of treatment for 20 days	Intrahippocampal KA-induced status epilepticus in mice	no effect on paroxysmal discharges in the hippocampus, inhibited granule cell dispersion and mossy fiber sprouting	[122]
		6 mg/kg/daily	For 3 weeks	electrical stimulation of angular bundle in rats	temporary remission of spontaneous seizures during treatment, seizures returned after the drug was discontinued	[123]
		3 mg/kg/daily	3 days prior to the induction of status epilepticus and continued for 3 weeks	electrical stimulation of angular bundle in rats	no effect on the development of spontaneous seizures	[123]
		3 mg/kg/daily	during the phase of spontaneous seizures for 5 days	electrical stimulation of angular bundle in rats	reduction of seizure frequency	[123]
		6 mg/kg/daily	for 1 week, then every second day for 6 weeks	a model of electrical stimulation of the angular bundle in rats	complete elimination of seizures in 25% of the animals and a significant reduction in their frequency in the remaining rats, neuroprotective effect in the hippocampus, a reduction in the permeability of the BBB	[124]
		6 mg/kg/daily	1 h after brain injury, continued once daily for up 4 weeks	a mouse model of traumatic brain injury	12.5% compared with 50% (control group) showed spontaneous electroencephalographic seizures, the study group had a frequency of seizures almost 10 times lower than the control group, inhibited neurodegeneration and mossy fiber sprouting	[125]
Apigenin *		50 mg/kg/daily, orally	for 6 days starting 5 days before status epilepticus	intracerebroventricular KA-induced status epilepticus in rats	reduced number of spontaneous epileptiform spikes	[126]
		50 mg/kg/daily	for six days following a status epilepticus	intracerebroventricular KA-induced status epilepticus in rats	a reduction in spontaneous epileptiform spikes, neurodegeneration, aberrant neurogenesis and mossy fiber sprouting, and m-TOR hyperactivity	[127]
Statins	Atorvastatin	10 mg/kg/daily	for 14 days, started 7 days prior to status epilepticus	a model of electrical stimulation of the angular bundle in rats	no effect on the development of spontaneous seizure activity, no effect on the permeability of the BBB, no effect on the loss of hilar cells or the regrowth of moss fibers	[139]
		10 or 100 mg/kg/daily, intragastrically	for 14 days	pilocarpine-induced status epilepticus in mice	no neuroprotection in the hilus of dentate gyrus, the response time to myoclonic jerks or tonic–clonic seizures has been reduced	[135]
		10 or 100 mg/kg/daily, intragastrically	for 14 days	pilocarpine-induced status epilepticus in mice	reduced inflammatory markers in the cortex and hippocampus, improvement in behavioral test results, an increase in the level of the anti-inflammatory interleukin-10	[136]
		10 mg/kg/daily, orally	for 17 weeks (starting at 45 days of age)	WAG/Rij genetic absence epilepsy rat model	reduced the development of absence seizures in rats at 6 months of age, reduced immobility time in the forced swimming test, and reduced anxiety in the open field test	[138]
	Simvastatin	10 mg/kg/daily, orally	for 17 weeks (starting at 45 days of age)	WAG/Rij genetic absence epilepsy rat model		[138]
	Pravastatin	30 mg/kg/daily, orally	for 17 weeks (starting at 45 days of age)	WAG/Rij genetic absence epilepsy rat model		[138]
	Lovastatin	20 mg/kg/twice/daily, intragastrically	via an esophagic probe 2 h after a status epilepticus onset and continued for 15 days	pilocarpine-induced status epilepticus in rats	significant decrease in the levels of inflammatory markers (IL-1β, TNF-α, and IL-6 during the latent phase and a decreased expression of IL-1β and TNF-α in the chronic chase), an increase in the level of the anti-inflammatory interleukin-10	[137]

*, not clinically approved; BBB, blood–brain barrier; KA, kainate.

## Data Availability

The data presented in this study are available in references number [1,2,3,4,5,6,7,8,9,10,11,12,13,14,15,16,17,18,19,20,21,22,23,24,25,26,27,28,29,30,31,32,33,34,35,36,37,38,39,40,41,42,43,44,45,46,47,48,49,50,51,52,53,54,55,56,57,58,59,60,61,62,63,64,65,66,67,68,69,70,71,72,73,74,75,76,77,78,79,80,81,82,83,84,85,86,87,88,89,90,91,92,93,94,95,96,97,98,99,100,101,102,103,104,105,106,107,108,109,110,111,112,113,114,115,116,117,118,119,120,121,122,123,124,125,126,127,128,129,130,131,132,133,134,135,136,137,138,139,140,141,142,143,144,145,146,147,148,149,150,151,152,153,154,155,156,157,158,159,160,161,162,163,164,165,166,167,168,169,170,171,172,173]. These data were derived from the following resources available in the public domain: PubMed (https://pubmed.ncbi.nlm.nih.gov (accessed on 7 April 2026)), Google Search (https://www.google.com), Web of Science (https://www.webofscience.com (accessed on 4 May 2026)).

## Abbreviations

ALK5	Activin-like kinase 5
AMPA	Alpha-amino-3-hydroxy-5-methyl- 4-isoxazolepropionic acid
AMPARs	AMPA receptors
anti-HMGB1 mAb	Anti-HMGB1 monoclonal antibody
ARE	Antioxidant response element
AQP4	Aquaporin-4
BBB	Blood–brain barrier
CAT	Catalase
CNS	Central nervous system
COX-2	Cyclooxygenase-2
CyP	Cyanobacterial product—selective antagonist of bacterial LPS
DZP	Diazepam
EEG	Electroencephalographic
GluR2	Glutamate receptor 2
GPx	Glutathione peroxidase
GR	Glutathione reductase
GSH	Reduced glutathione
GS-Pro	Glutathionylated proteins
GSSG	Glutathione disulfide
HMGB1	High-mobility group box-1
ICE	IL-1β converting enzyme
i.p.	Intraperitoneal
IGF-1	Insulin-like growth factor 1
IL-1R1	IL-1β receptor type 1
IL-1Ra	Interleukin-1 receptor antagonist
IL-1β	Interleukin-1β
IL-6	Interleukin-6
iNOS	Inducible nitric oxide synthase
JAK/STAT	Janus kinase/signal transducers and activators of transcription
KA	Kainate
Kir 4.1	Inward rectifying potassium
LPO	Lipid peroxidation
LPS	Lipopolysaccharide
mAb	Monoclonal antibody
MDA	Malondialdehyde
MLR	Monocyte-to-lymphocyte ratio
MS	Multiple sclerosis
mTOR	Mammalian target of rapamycin
NADPH	Nicotinamide adenine dinucleotide phosphate
NF-κB	Nuclear factor kappa-light-chain-enhancer of activated B cells
NLR	Neutrophil-to-lymphocyte ratio
NO	Nitric oxide
NOS	Nitric oxide synthase
NOX	NADPH oxidase
Nrf2	Nuclear erythroid-2-related factor 2 Pilo pilocarpine
OVLT	Organum vasculosum laminae terminalis
p38 MAPK	p38 mitogen-activated protein kinase
PGG2	Prostaglandin G2
PGI2	Prostacyclin I2
PGs	Prostaglandins
PI3K	Phosphatidylinositol 3-kinase
PILO	Pilocarpine
Prxs	Peroxiredoxins
PST	Posttraumatic epilepsy
PTZ	Pentylenetetrazol
Ra	Receptor antagonist
RAGE	Receptor for advanced glycation end products
RNS	Reactive nitrogen species
s.c.	Subcutaneous
SE	Status epilepticus
SOD	Supeoxide dismutase
SRS	Spontaneous recurrent seizure
TBI	Traumatic brain injury
TβRI	TGF-βI receptors
TβRII	TGF-β binds to receptor II
TGF-β	Transforming growth factor β
TLE	Temporal lobe epilepsy
TLR4	Toll-like receptor 4
TNF-α	Tumor necrosis factor-α.
TRPV1	Transient receptor potential vanilloid 1
TxA2	Thromboxane A2
TXN2	Thioredoxin 2
VX-765	Caspase-1 inhibitor
